# Supplementary figures and images for: Synthesis and characterization of cerium doped NiZn nano ferrites as substrate material for multi band MIMO antenna
Source: PLoS One. 2024 Jul 16;19(7):e0305060. doi: 10.1371/journal.pone.0305060 (PMC11251584; doi:10.1371/journal.pone.0305060)

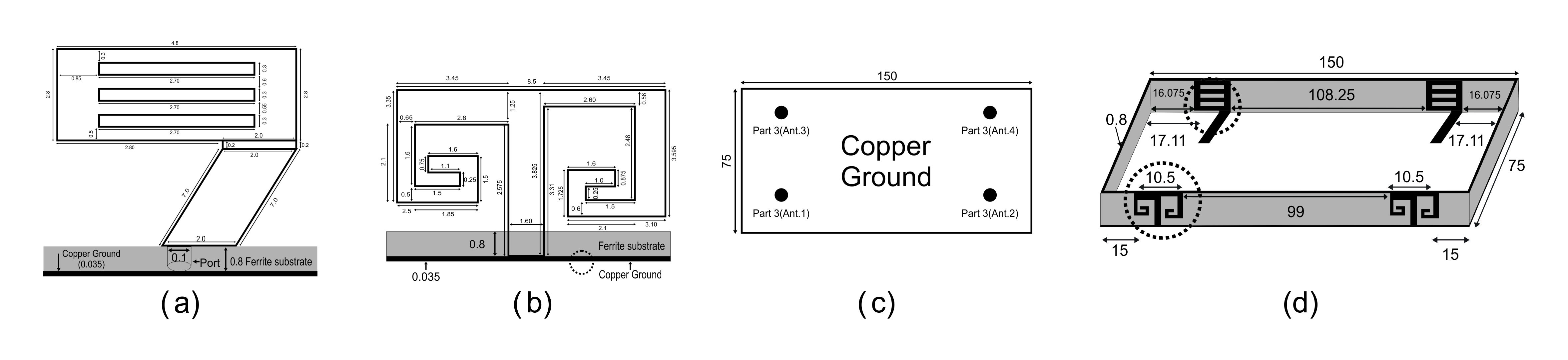

Supplement: S1 File — (ZIP) [file pone.0305060.s001.zip › supplementary information files/ANTINA DESIGN 2.jpg]

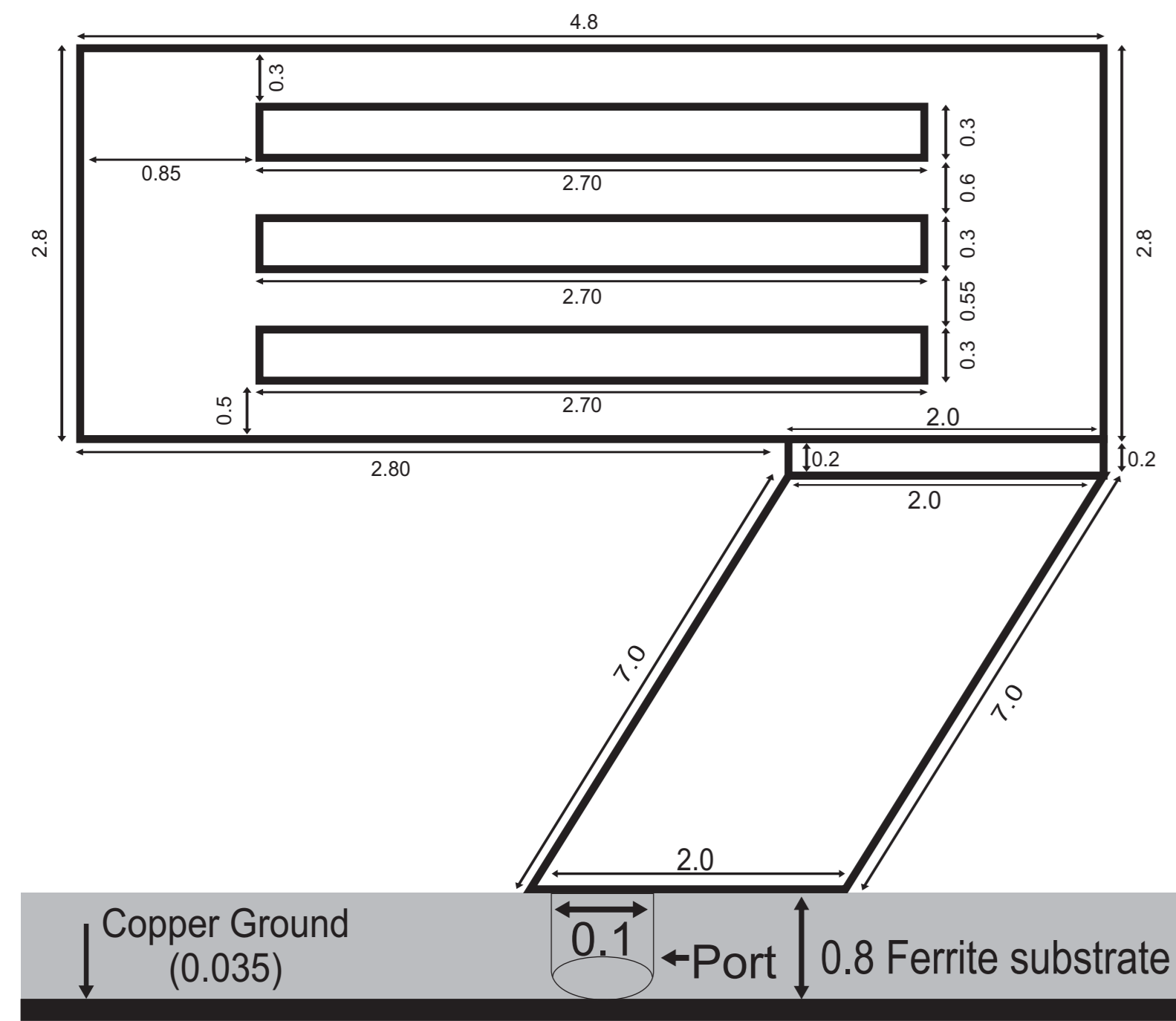

(a)

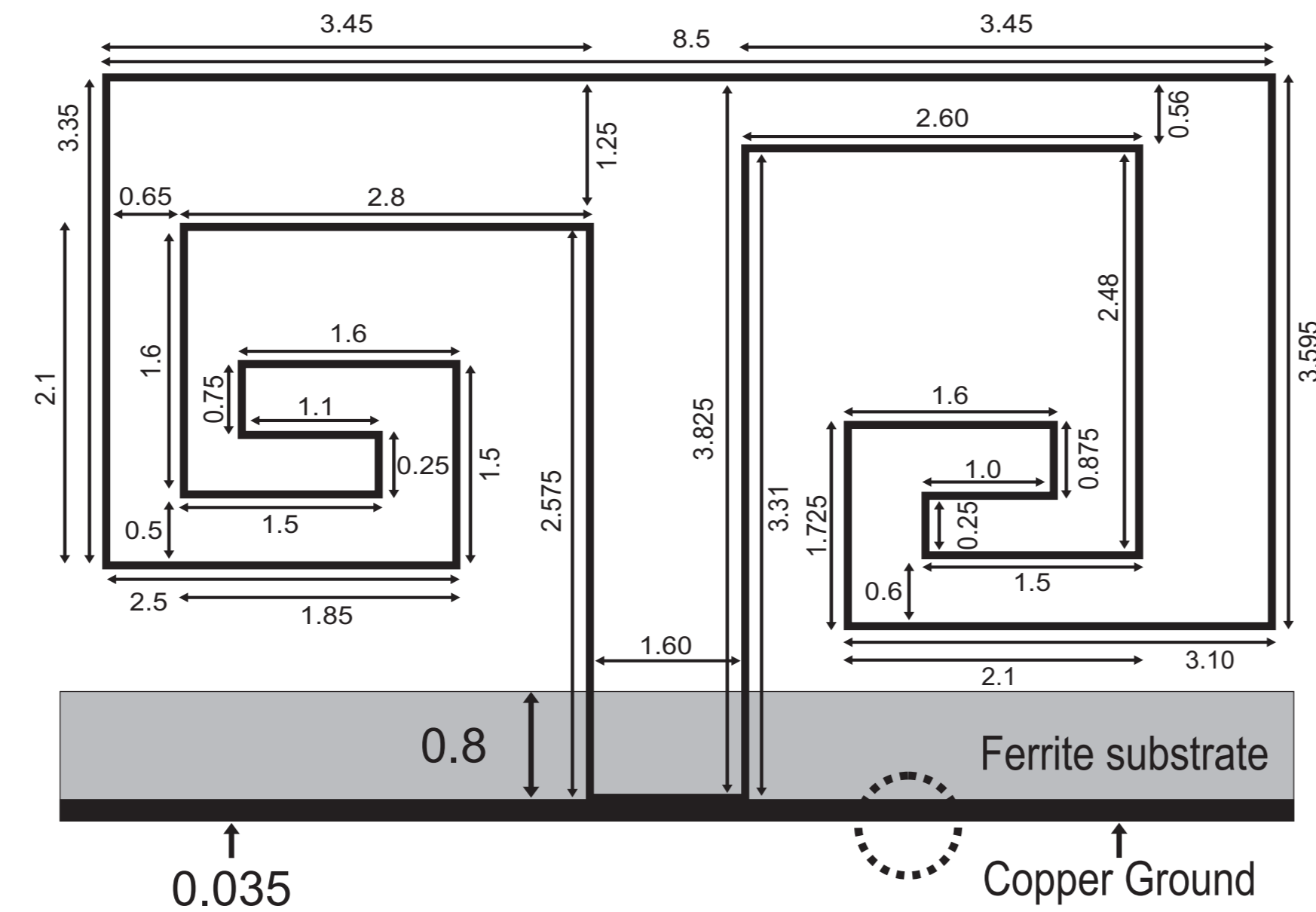

(b)

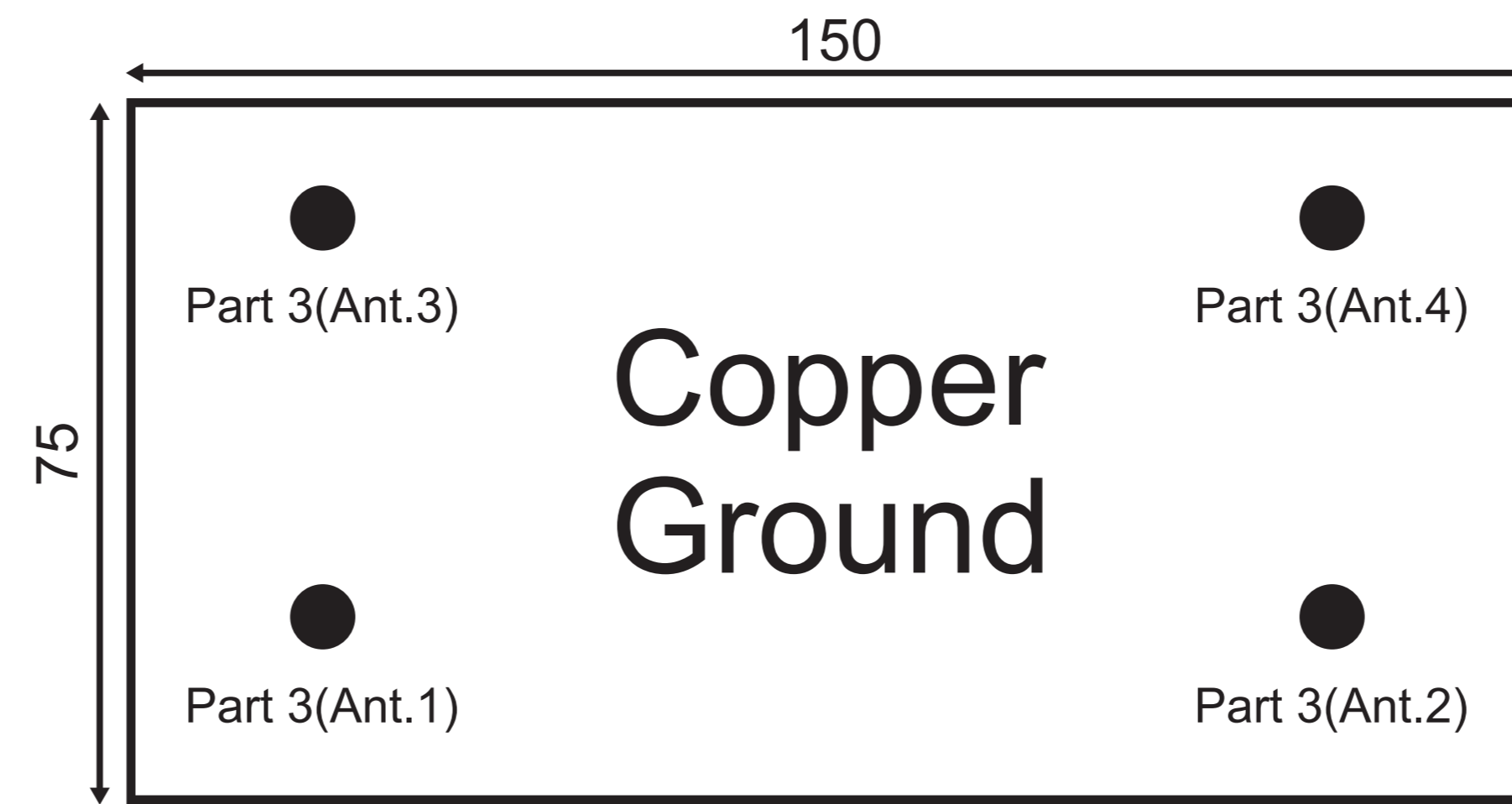

(c)

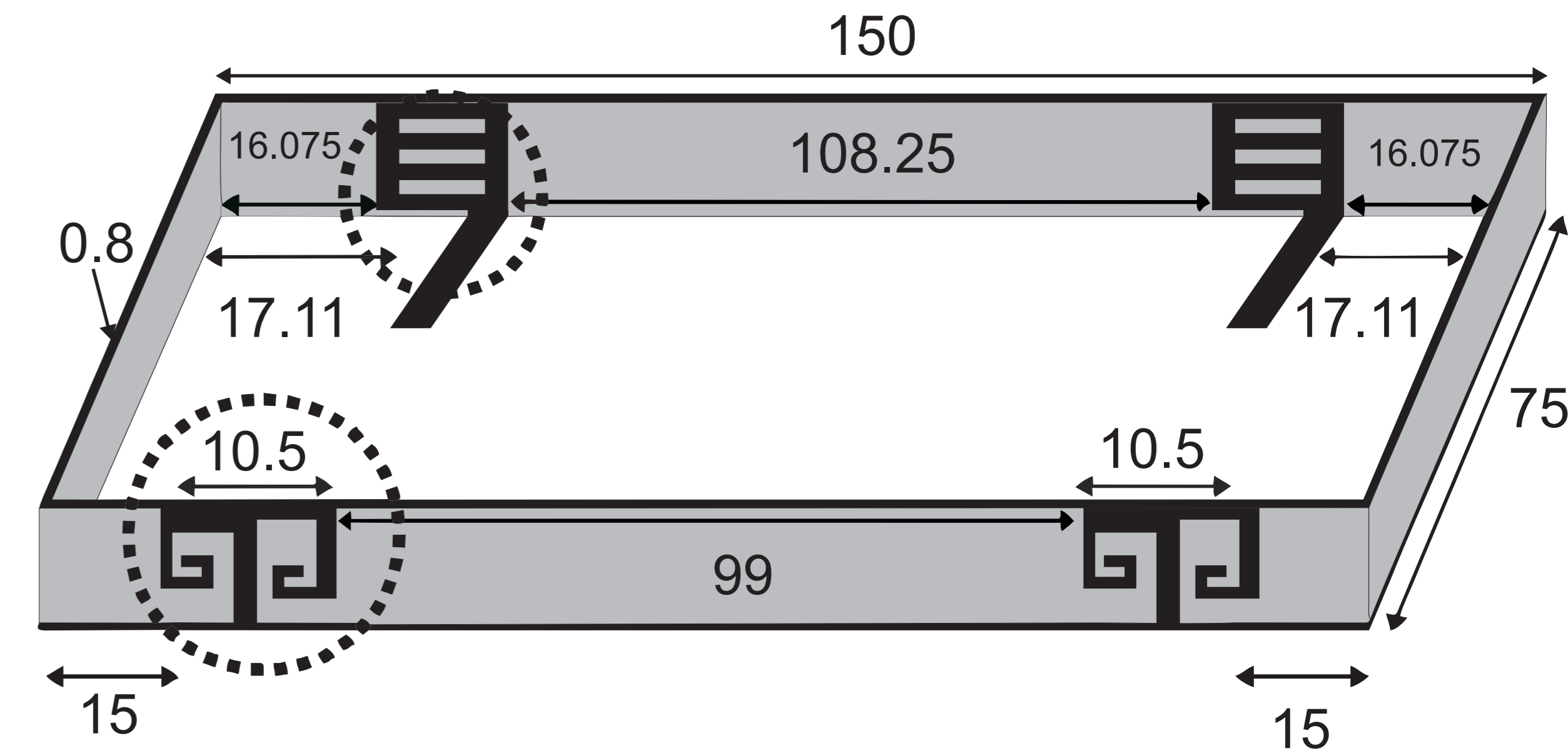

(d)

Supplement: S1 File — (ZIP) [file pone.0305060.s001.zip › supplementary information files/ANTINA DESIGN 2.pdf]

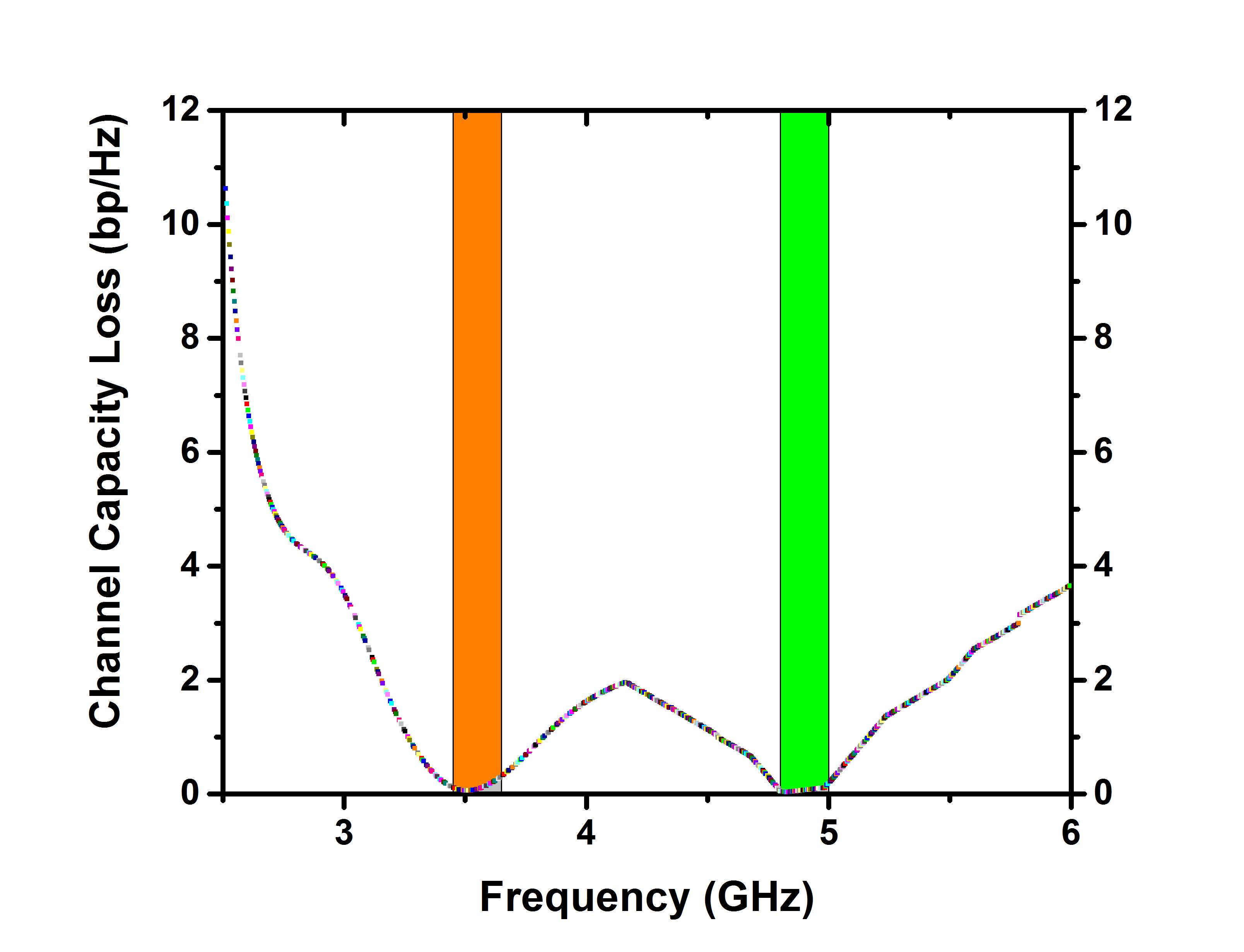

Supplement: S1 File — (ZIP) [file pone.0305060.s001.zip › supplementary information files/CCL/CCL.jpg]

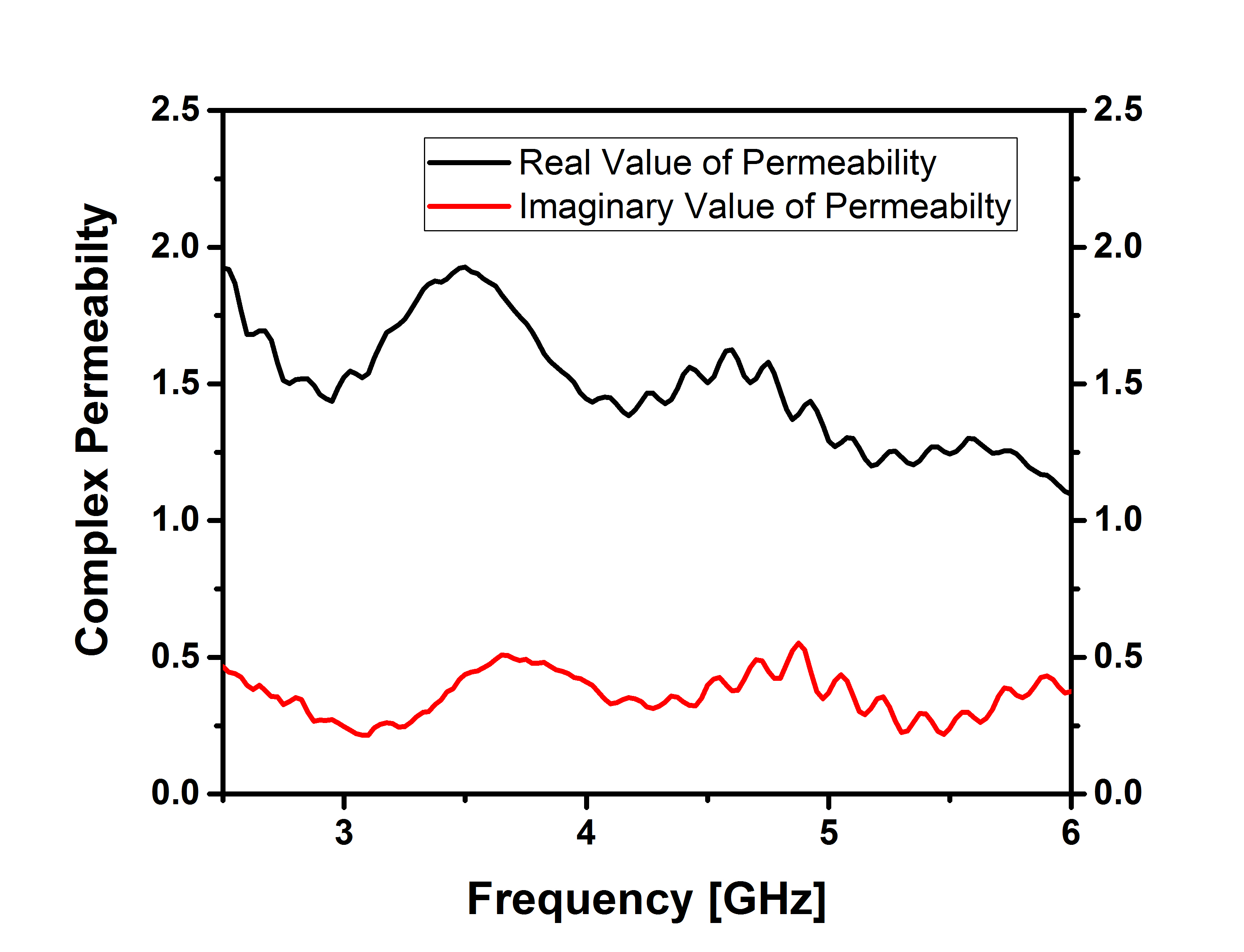

Supplement: S1 File — (ZIP) [file pone.0305060.s001.zip › supplementary information files/Dielectric Properties/complex permeability.jpg]

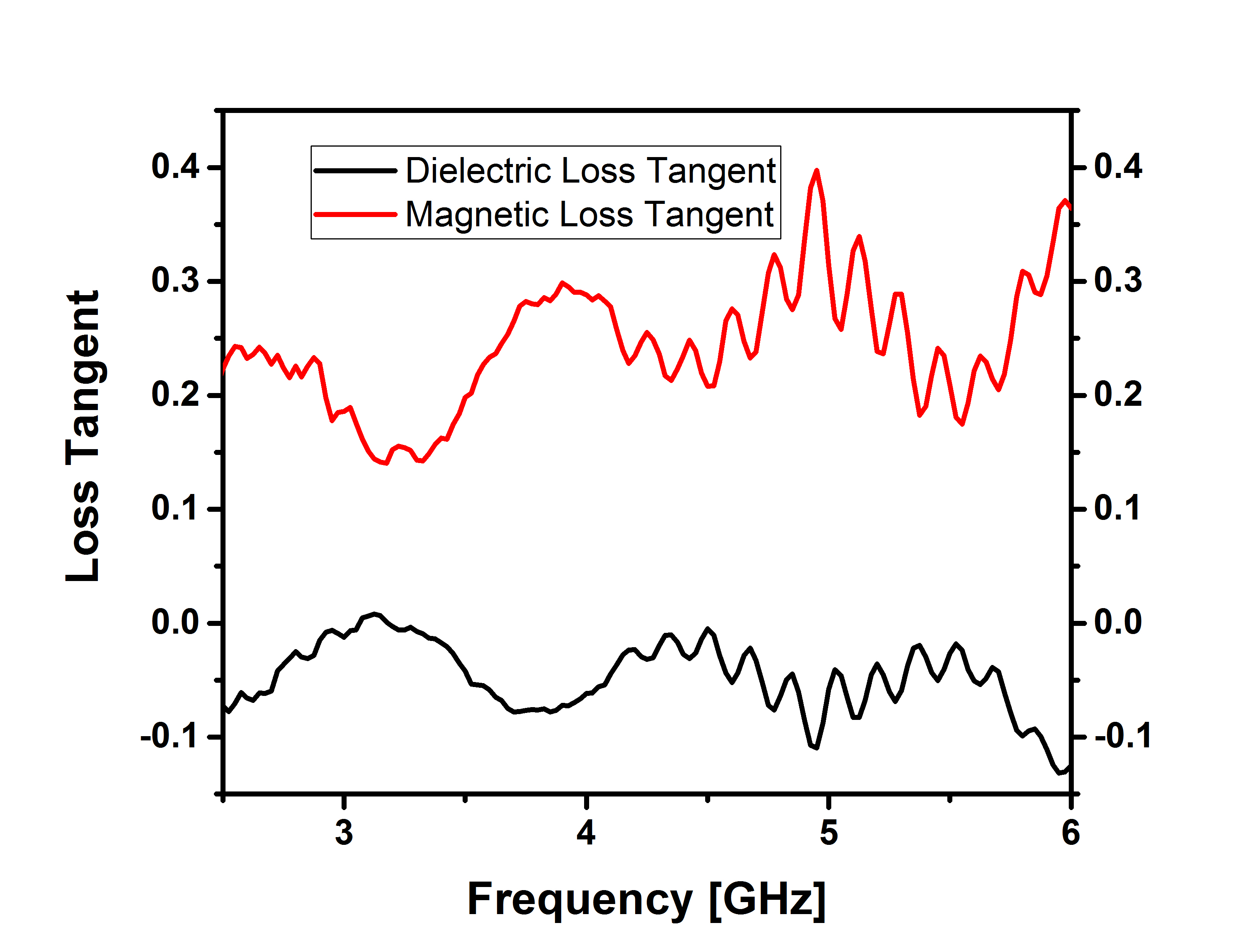

Supplement: S1 File — (ZIP) [file pone.0305060.s001.zip › supplementary information files/Dielectric Properties/Loss tangent.jpg]

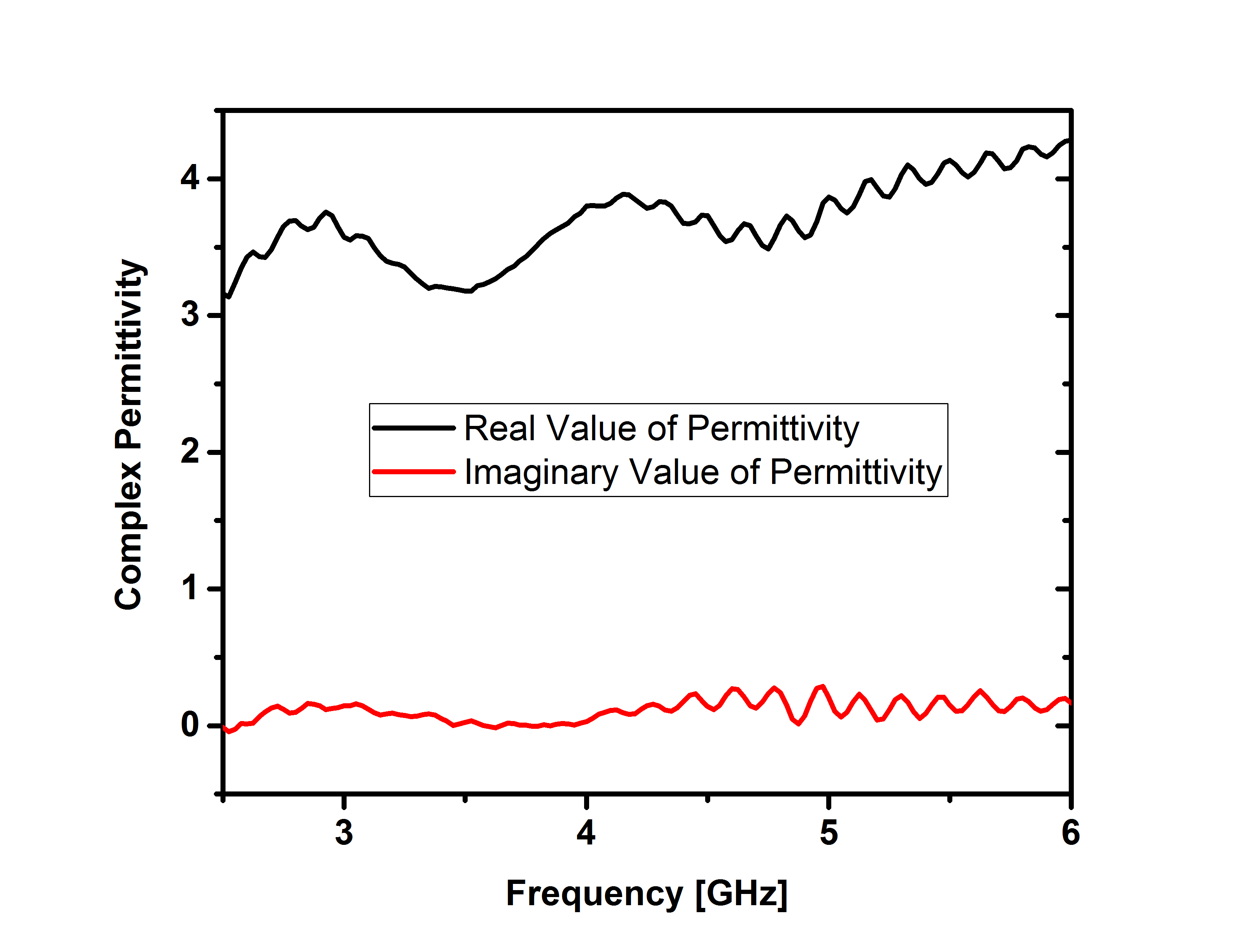

Supplement: S1 File — (ZIP) [file pone.0305060.s001.zip › supplementary information files/Dielectric Properties/permittivity for 2nd paper.jpg]

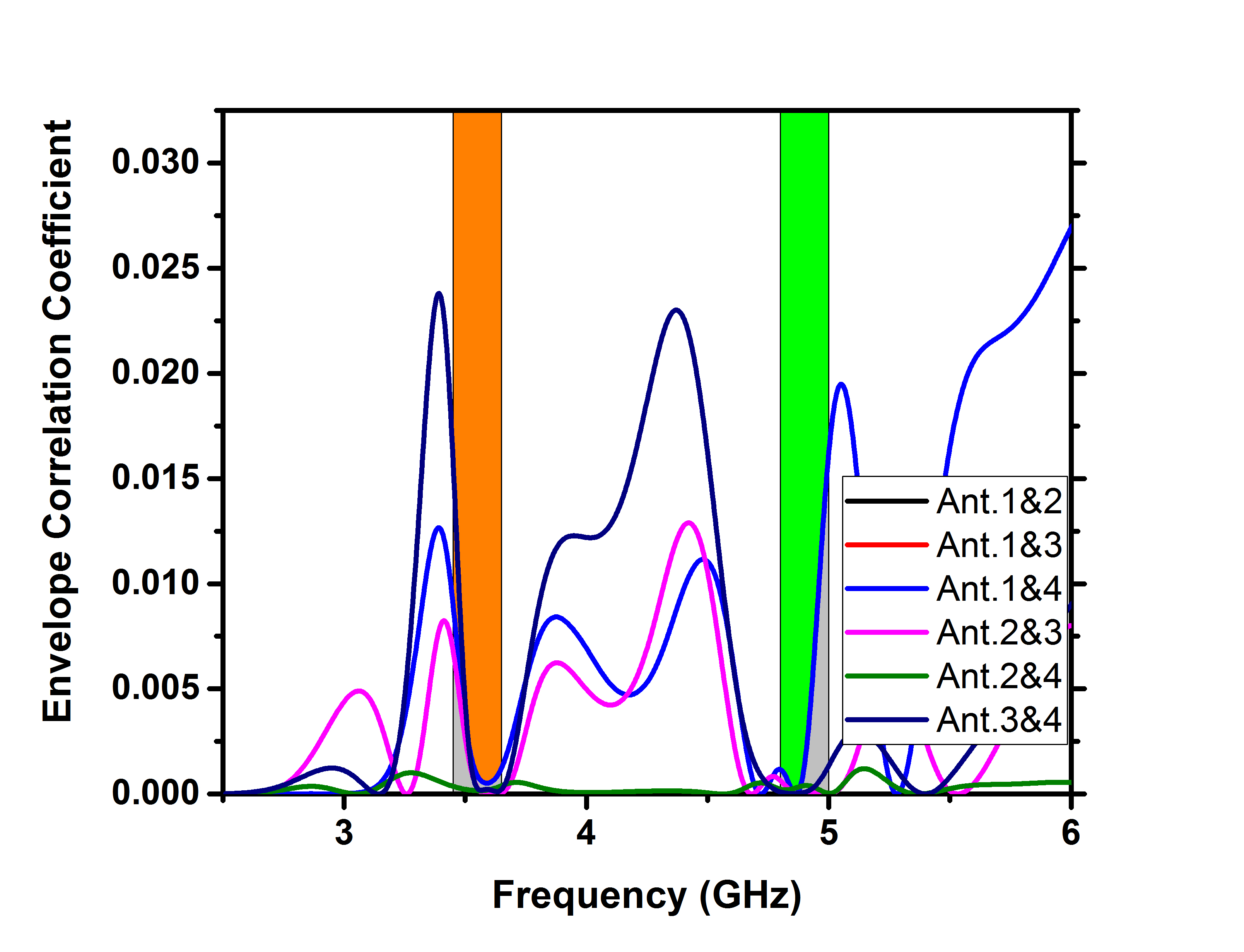

Supplement: S1 File — (ZIP) [file pone.0305060.s001.zip › supplementary information files/ECC/ECC.jpg]

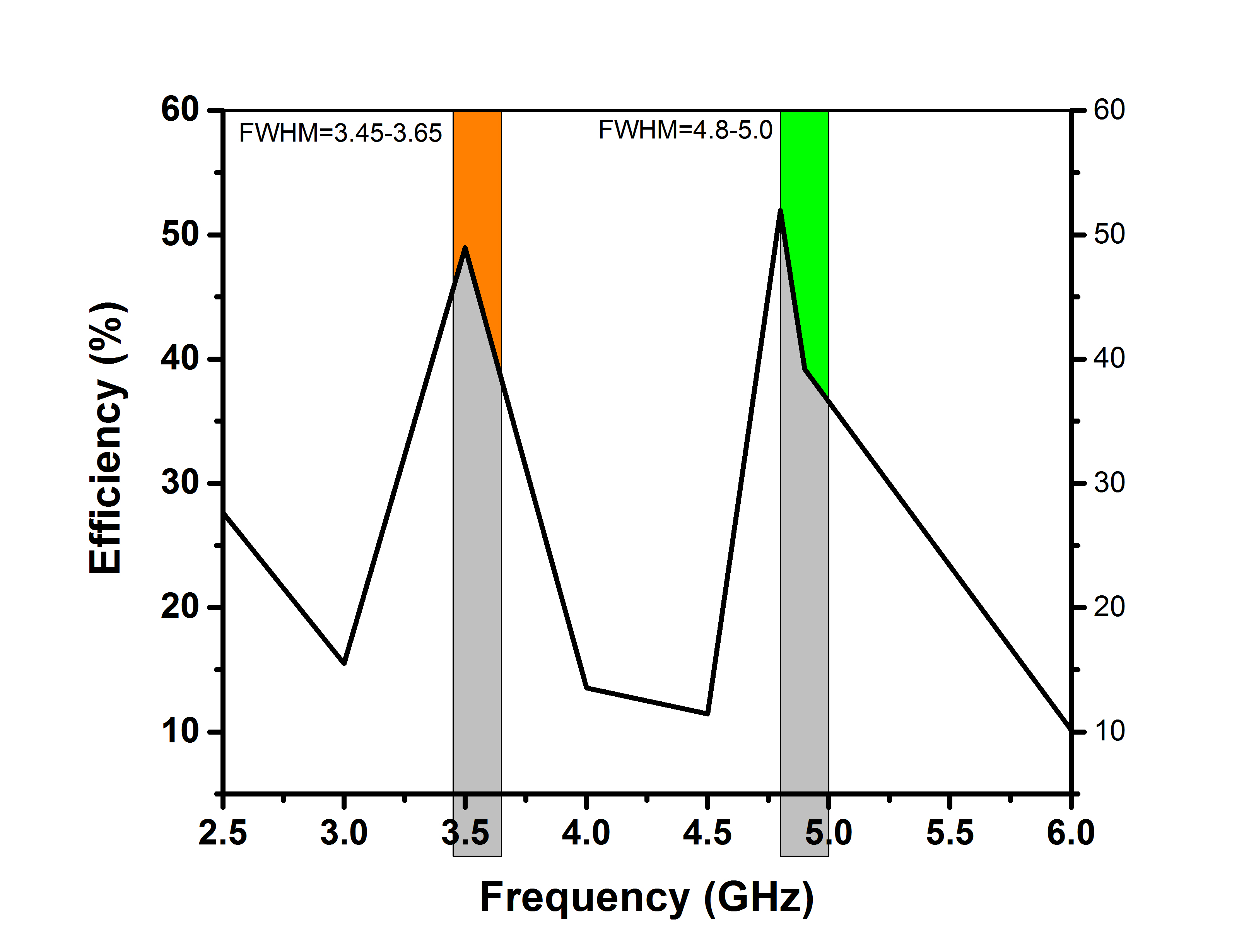

Supplement: S1 File — (ZIP) [file pone.0305060.s001.zip › supplementary information files/Efficiency/efficiency.jpg]

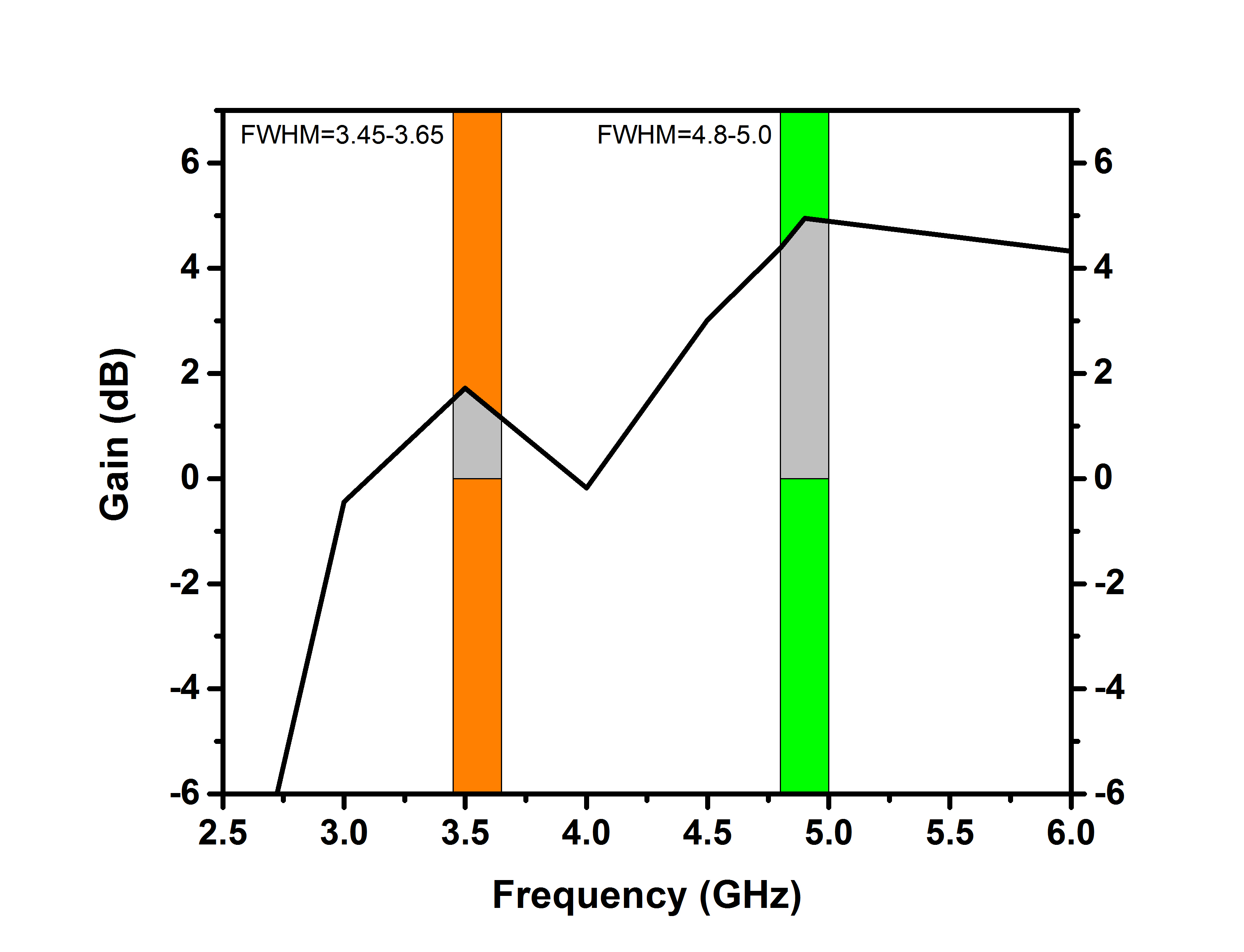

Supplement: S1 File — (ZIP) [file pone.0305060.s001.zip › supplementary information files/Gain/gain.jpg]

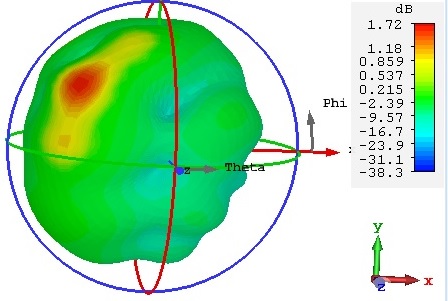

Supplement: S1 File — (ZIP) [file pone.0305060.s001.zip › supplementary information files/Gain/pic 1.jpg]

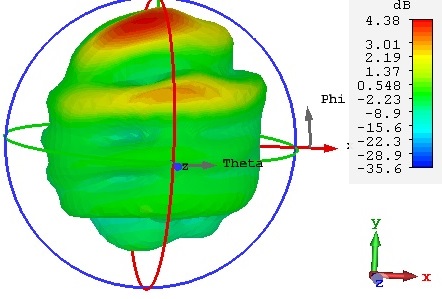

Supplement: S1 File — (ZIP) [file pone.0305060.s001.zip › supplementary information files/Gain/pic 2.jpg]

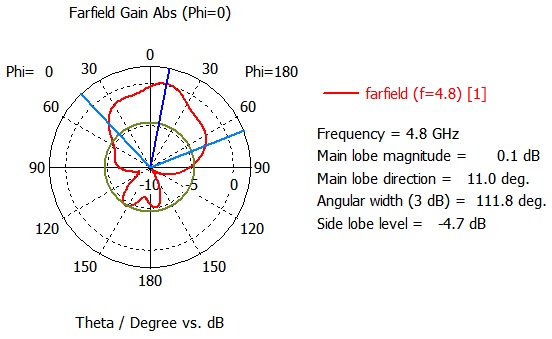

Supplement: S1 File — (ZIP) [file pone.0305060.s001.zip › supplementary information files/Gain/pic 3.jpg]

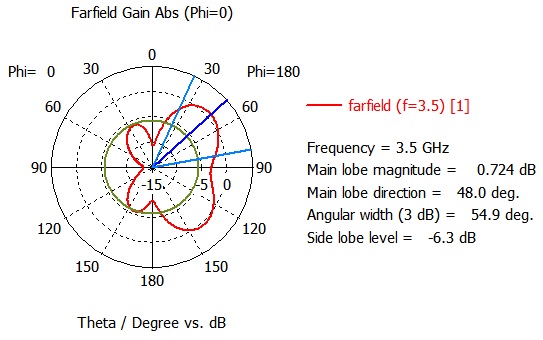

Supplement: S1 File — (ZIP) [file pone.0305060.s001.zip › supplementary information files/Gain/pic 4.jpg]

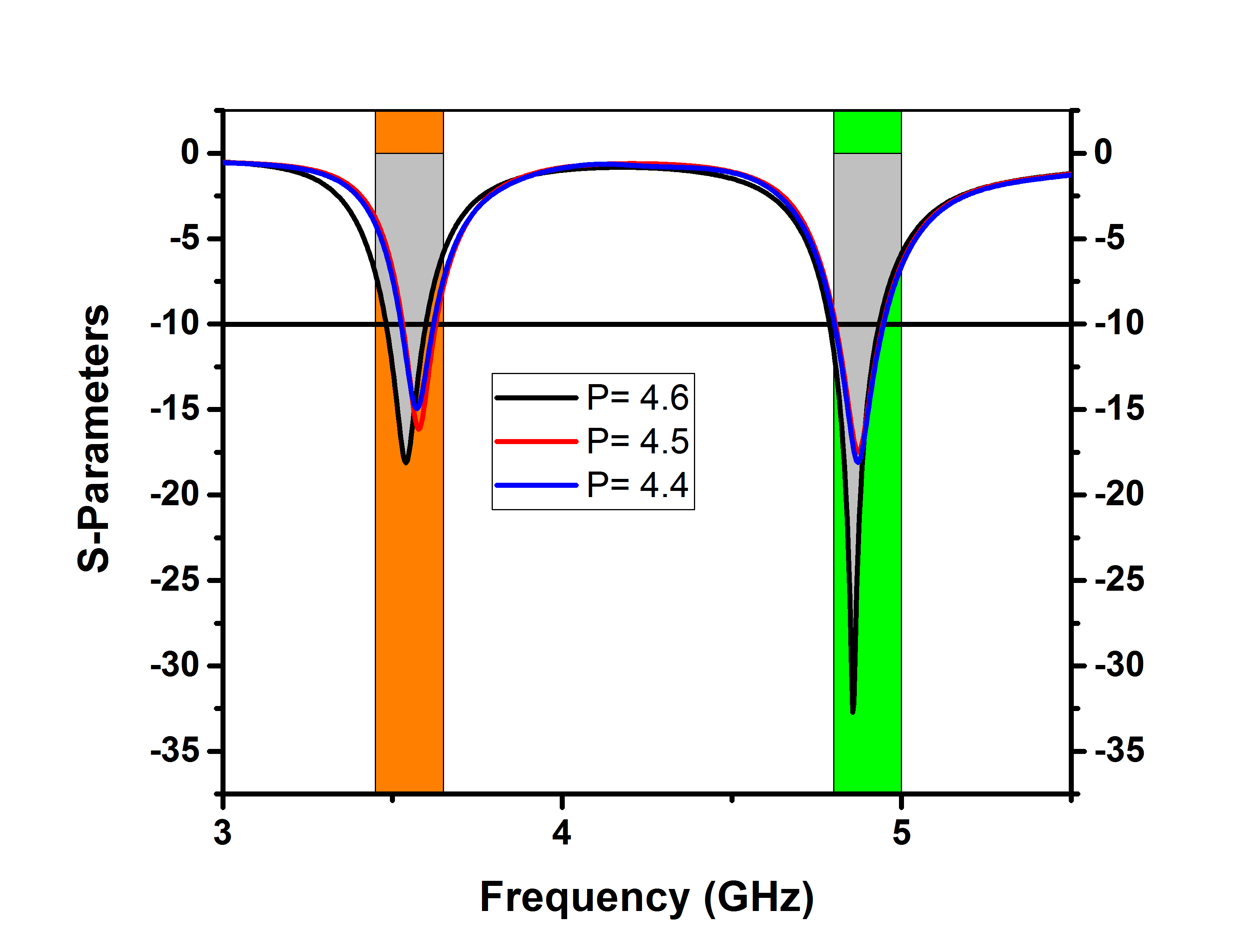

Supplement: S1 File — (ZIP) [file pone.0305060.s001.zip › supplementary information files/Parametric Analysis/effect of P on graph/effect of P on S parameters.jpg]

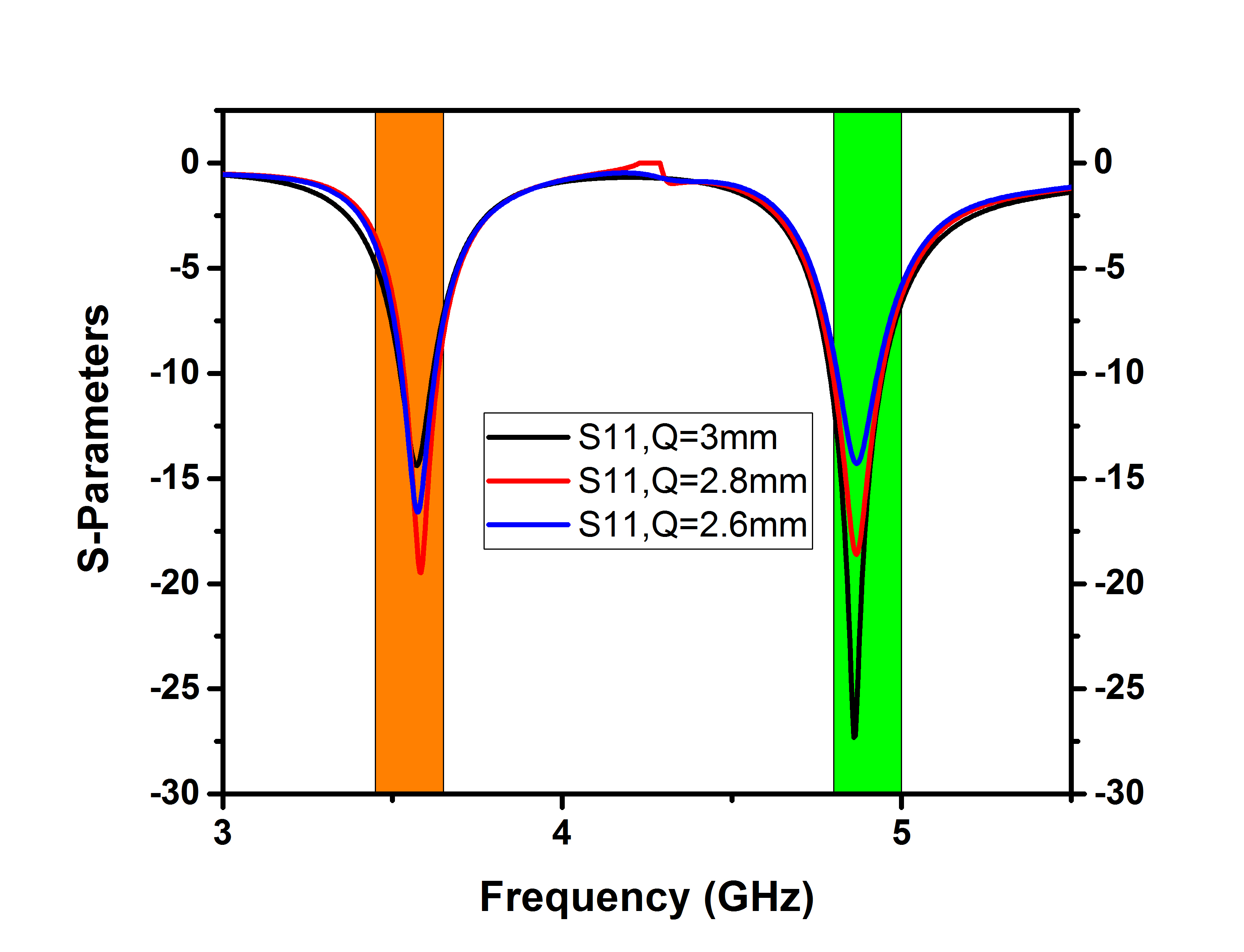

Supplement: S1 File — (ZIP) [file pone.0305060.s001.zip › supplementary information files/Parametric Analysis/effect of Q/When Q changes.jpg]

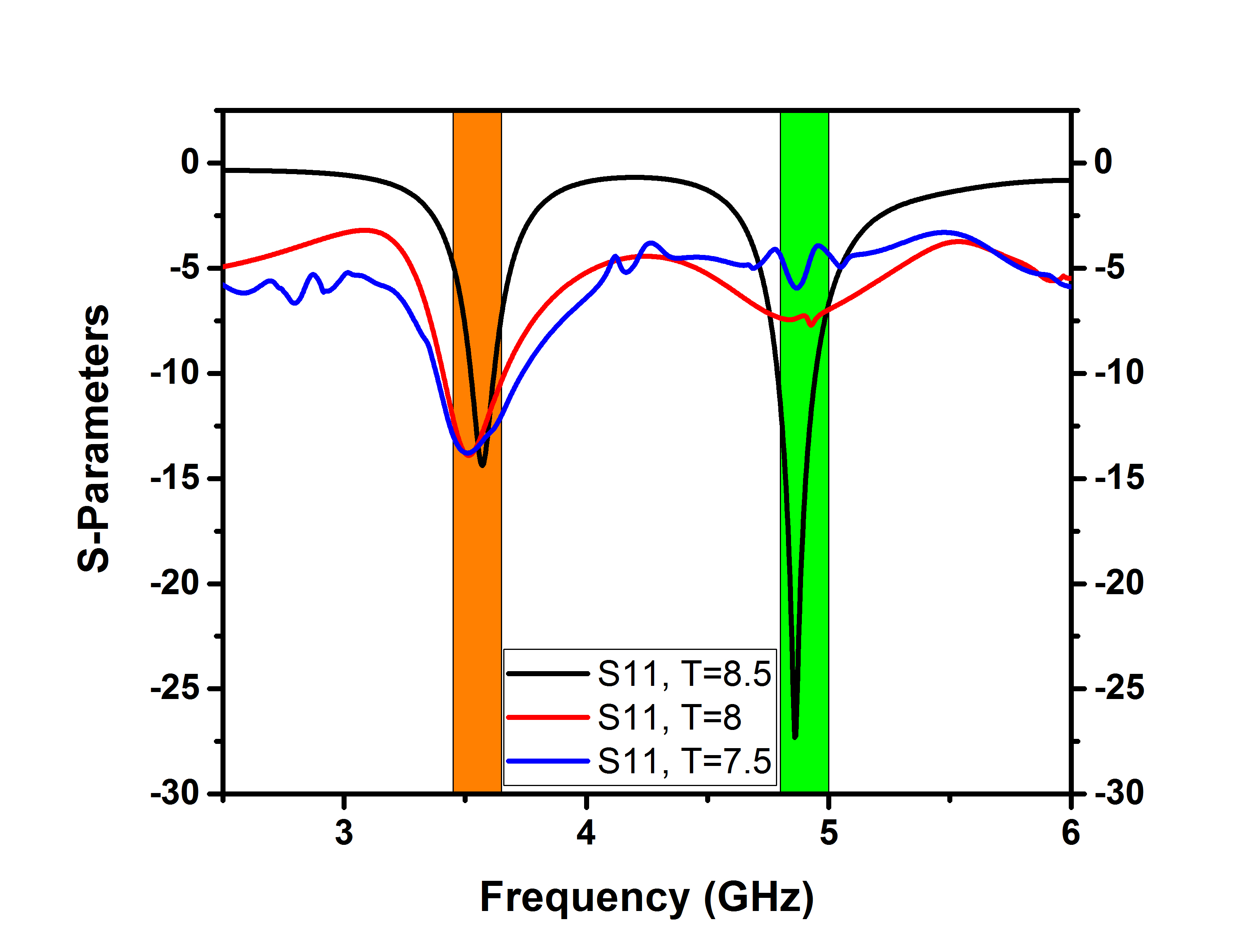

Supplement: S1 File — (ZIP) [file pone.0305060.s001.zip › supplementary information files/Parametric Analysis/effect of T/variation with T.jpg]

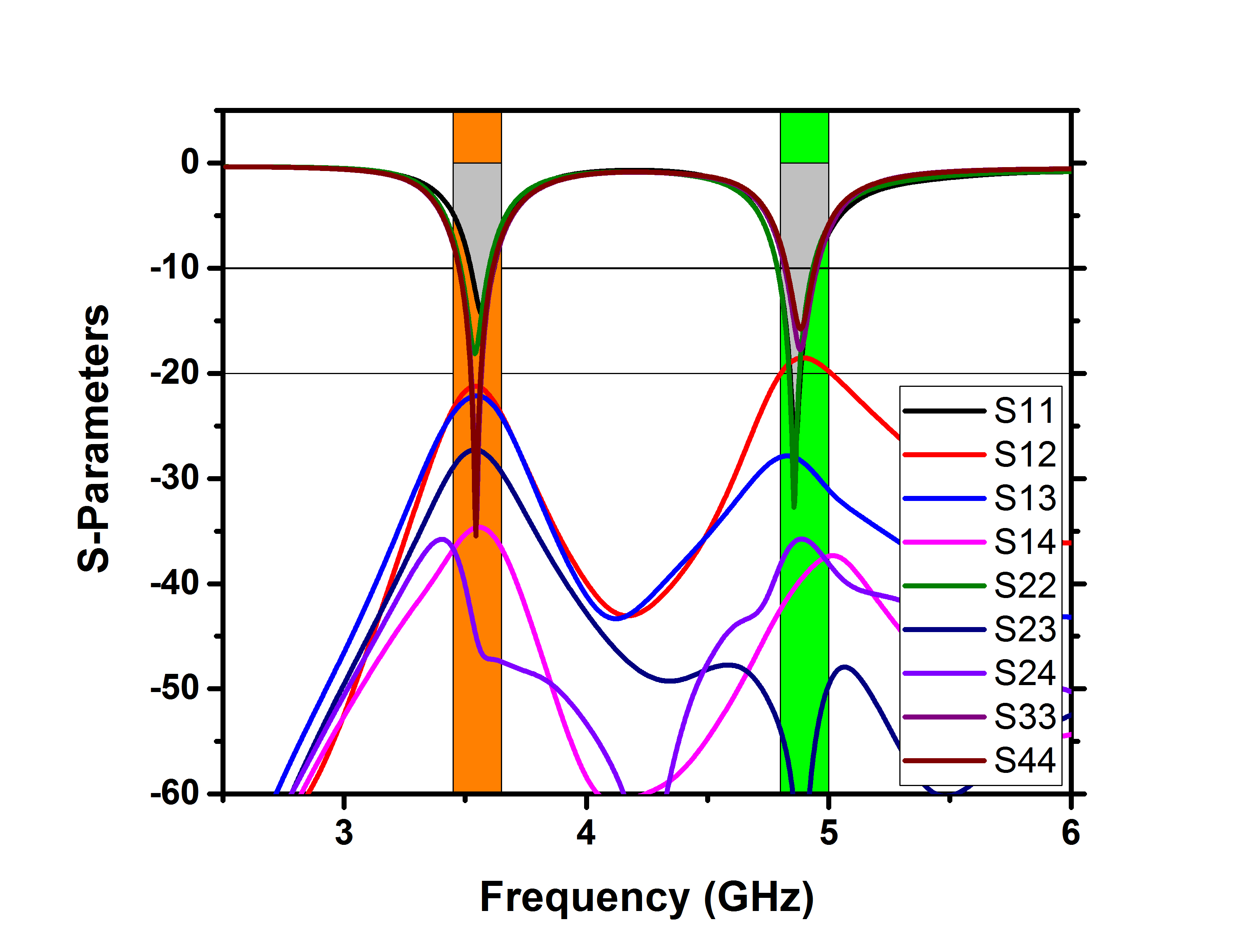

Supplement: S1 File — (ZIP) [file pone.0305060.s001.zip › supplementary information files/S-parameters/S-parameters.jpg]

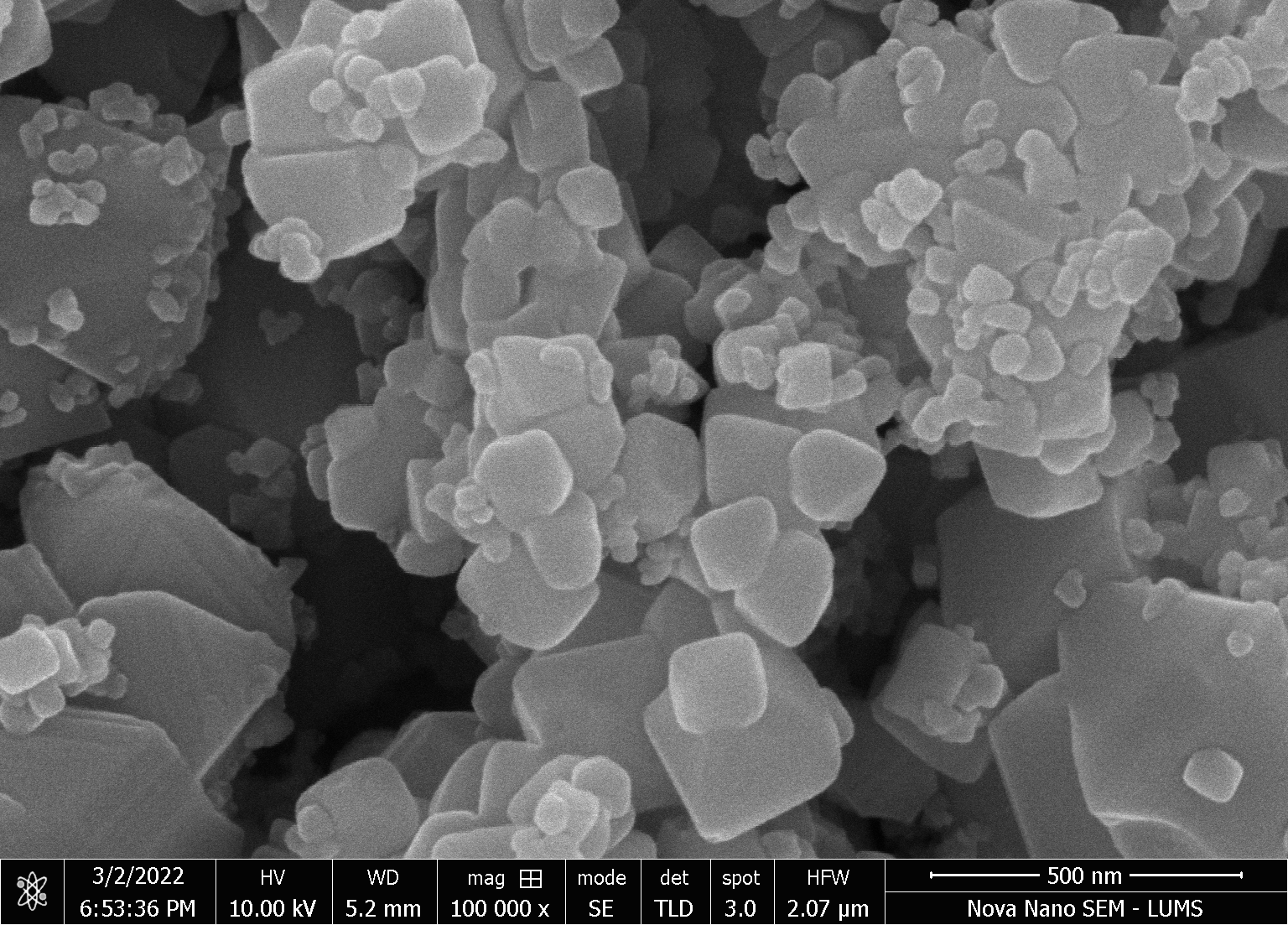

Supplement: S1 File — (ZIP) [file pone.0305060.s001.zip › supplementary information files/SEM/SEM result.tif]

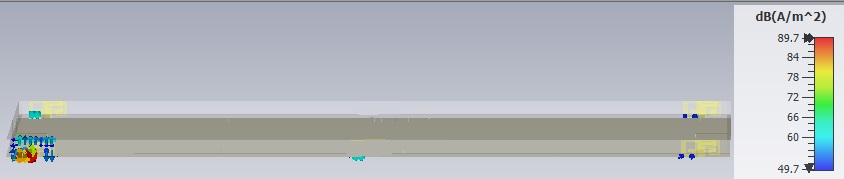

Supplement: S1 File — (ZIP) [file pone.0305060.s001.zip › supplementary information files/surface current/All 4 antennas.jpg]

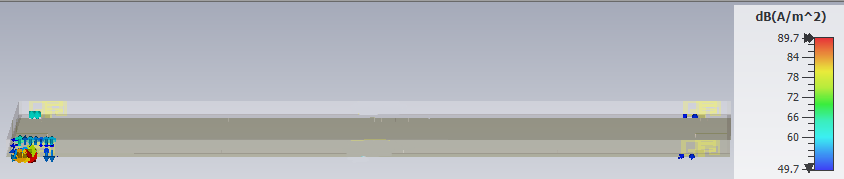

Supplement: S1 File — (ZIP) [file pone.0305060.s001.zip › supplementary information files/surface current/All 4 antennas.png]

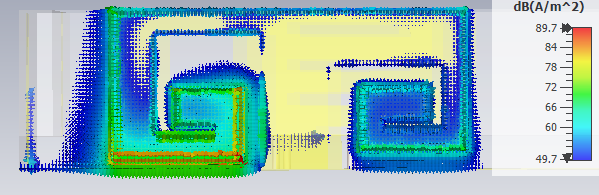

Supplement: S1 File — (ZIP) [file pone.0305060.s001.zip › supplementary information files/surface current/Antenna 1.png]

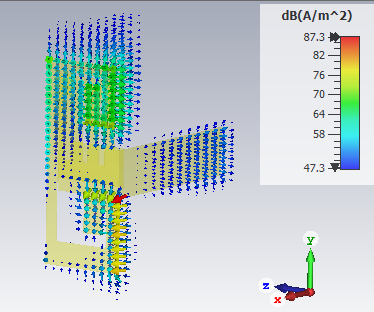

Supplement: S1 File — (ZIP) [file pone.0305060.s001.zip › supplementary information files/surface current/picture a.png]

## Slide 1
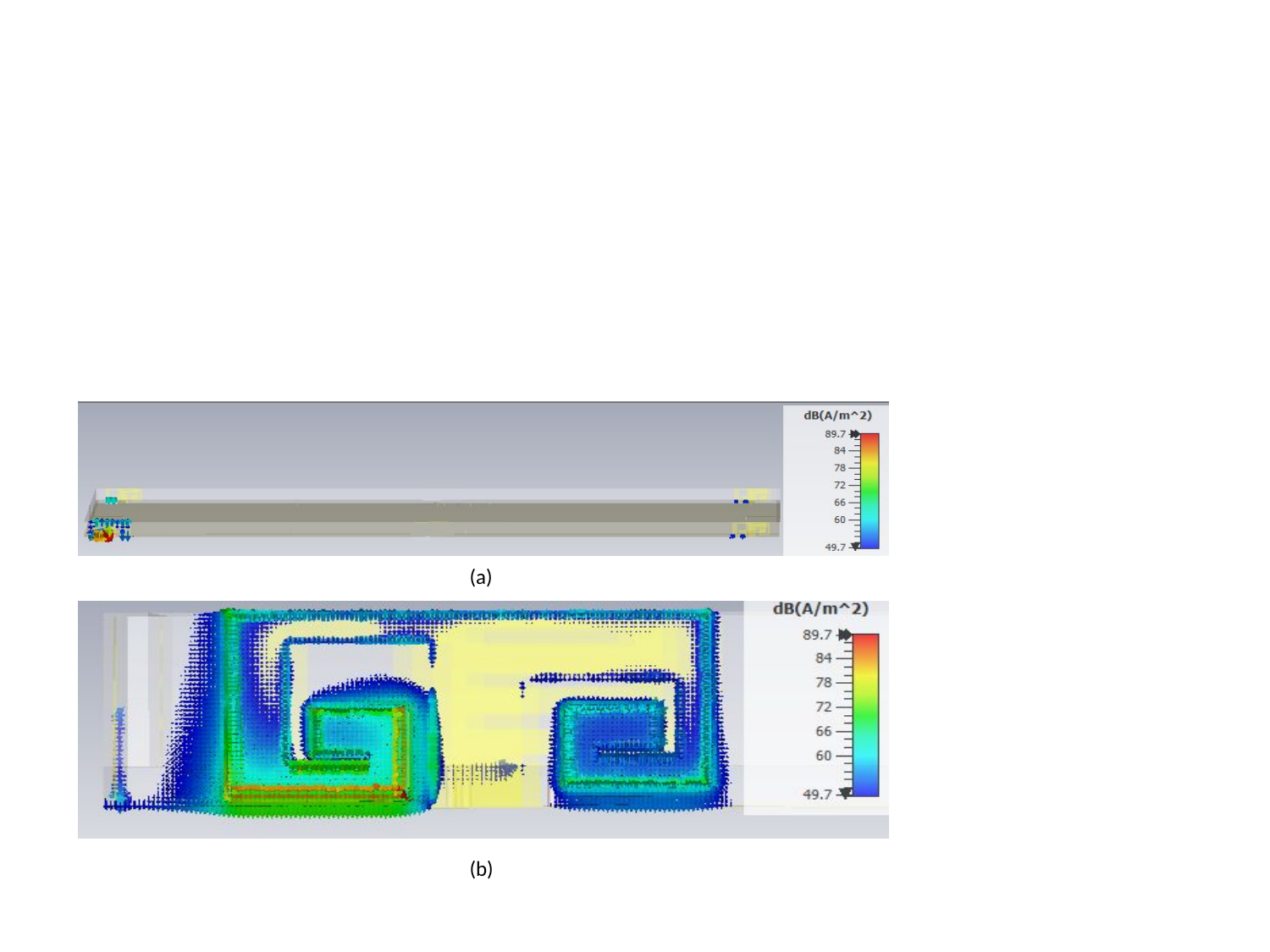

(a)
(b)

## Slide 2
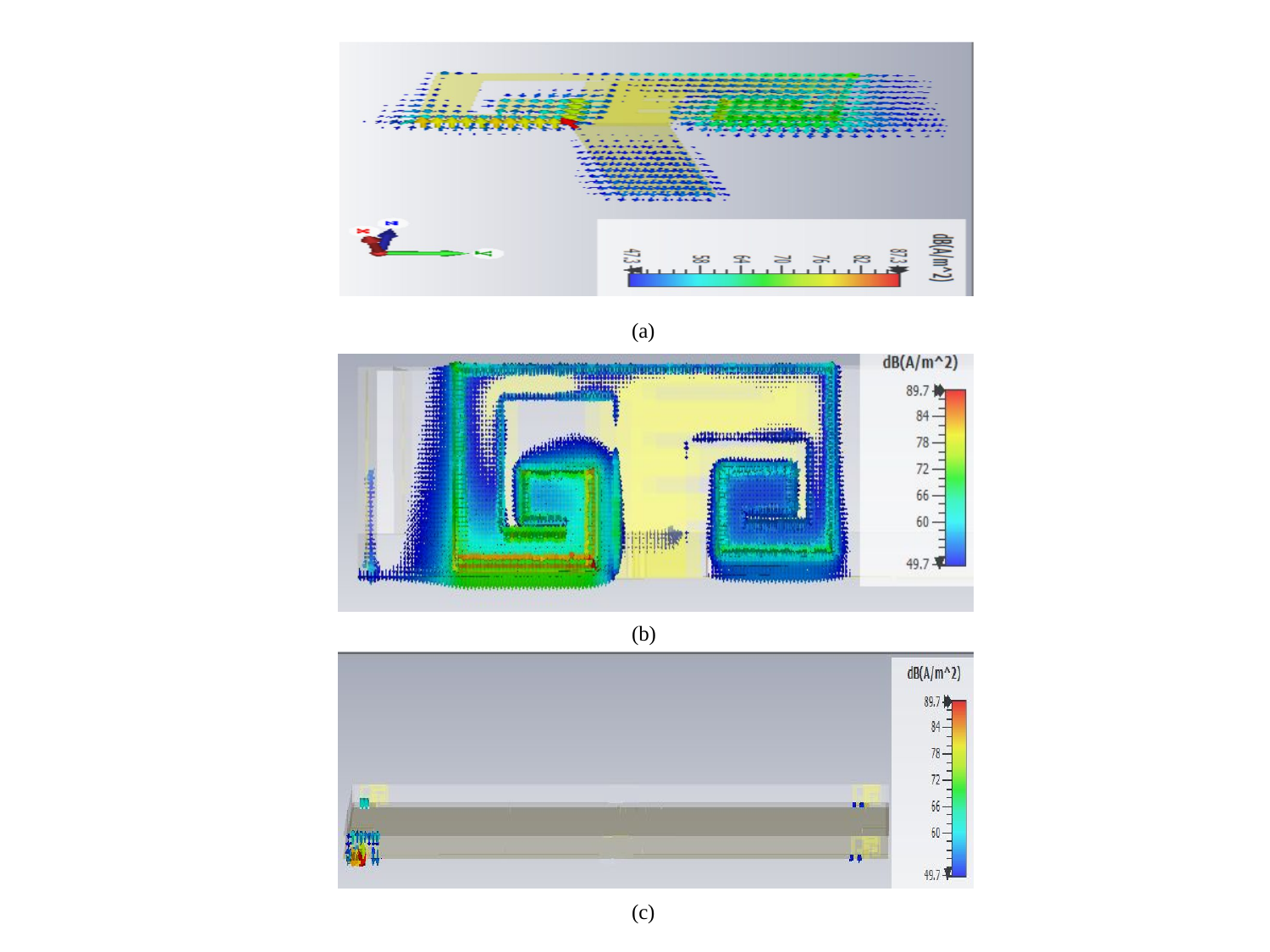

(a)
(b)
(c)

## Slide 3
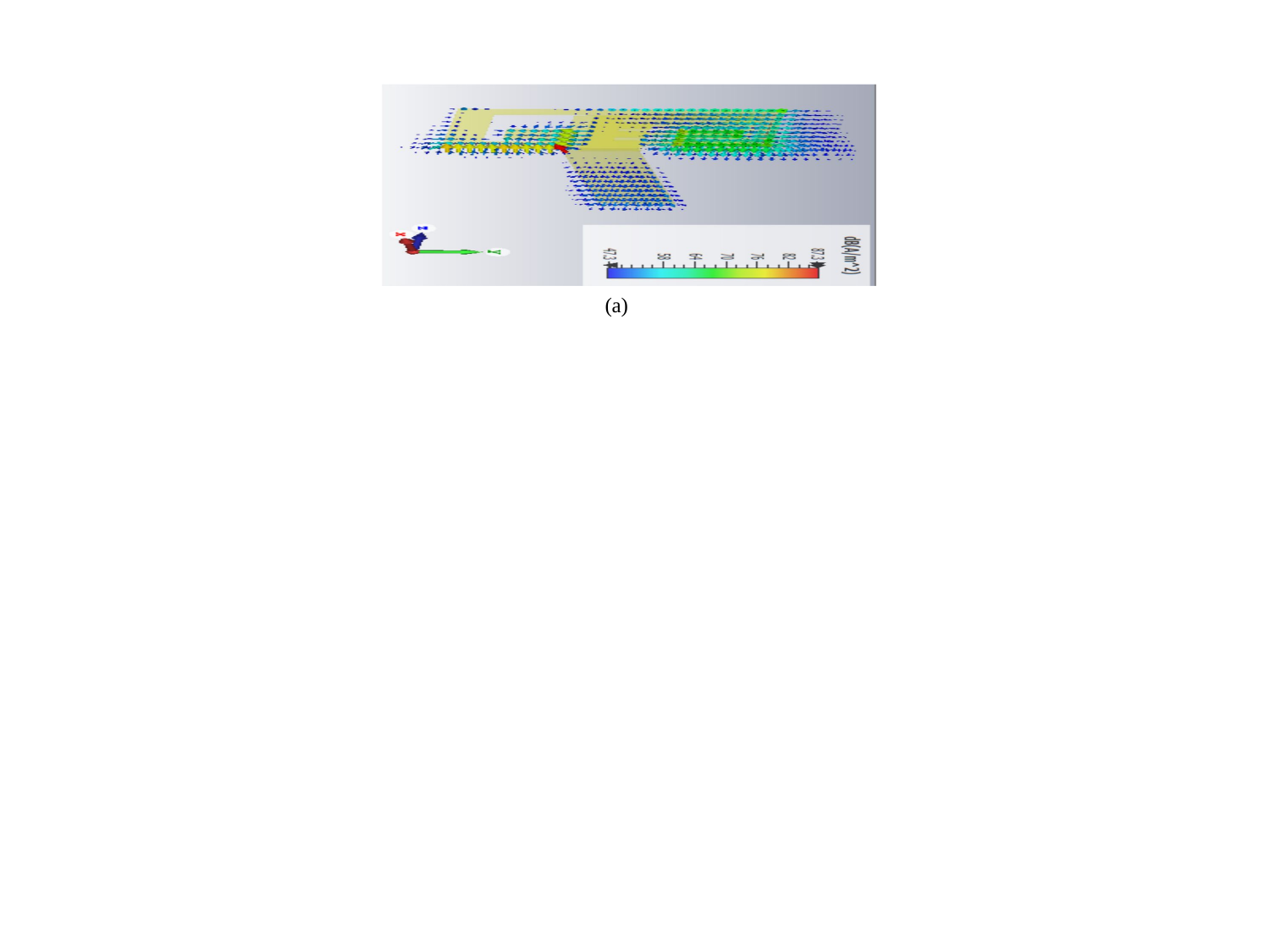

(a)

Supplement: S1 File — (ZIP) [file pone.0305060.s001.zip › supplementary information files/surface current/Presentation1.pptx]

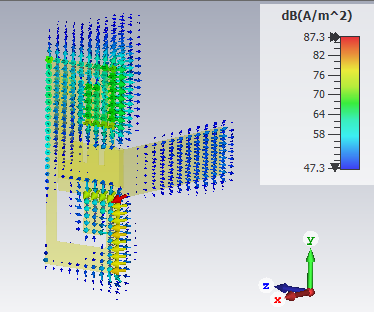

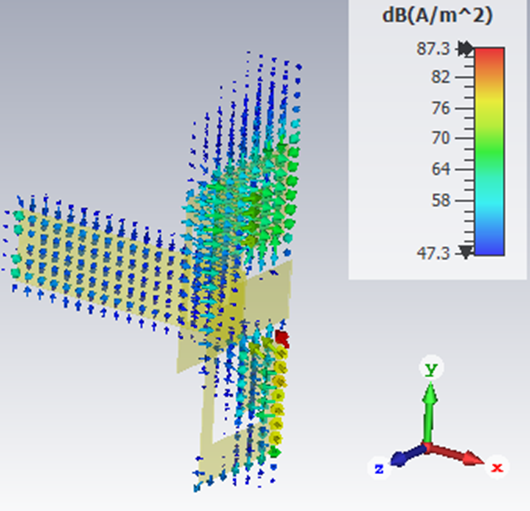


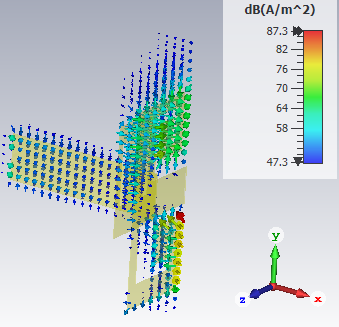


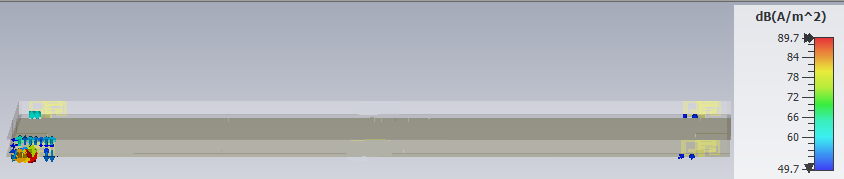

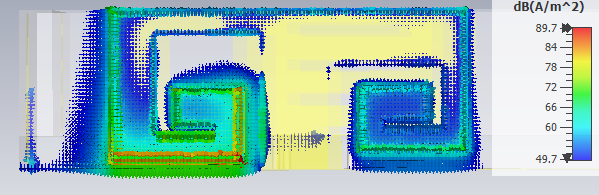


(a)

(b)


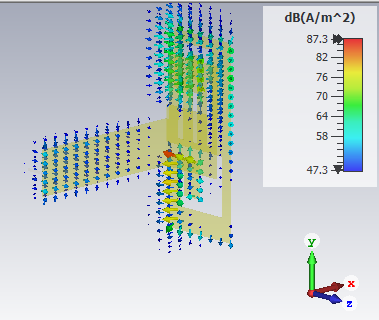


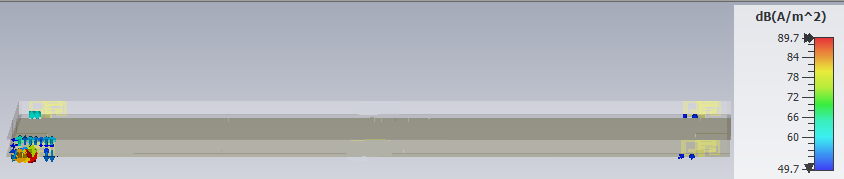

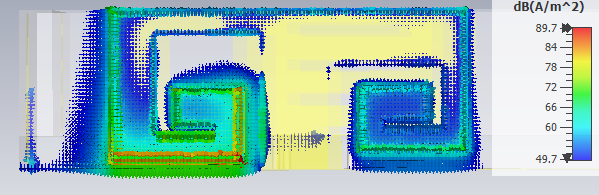


(a)

(b)


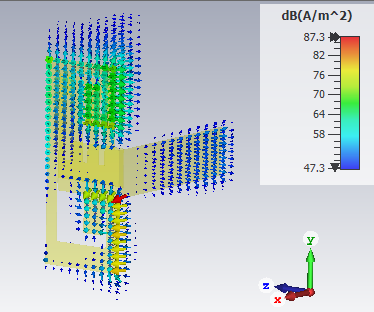


(a)


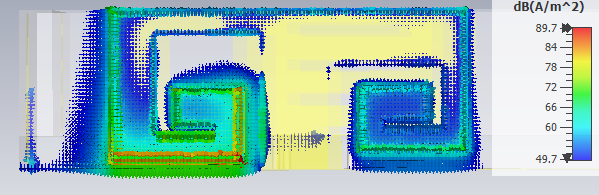


(b)


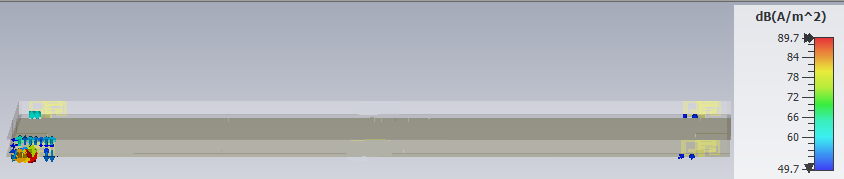


(c)


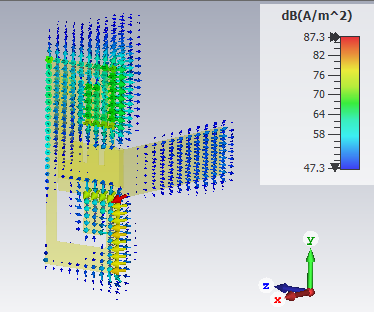


(c)


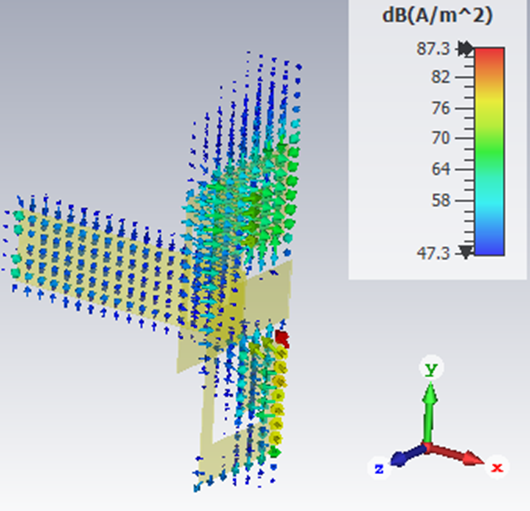

Supplement: S1 File — (ZIP) [file pone.0305060.s001.zip › supplementary information files/surface current/raeesb.docx]

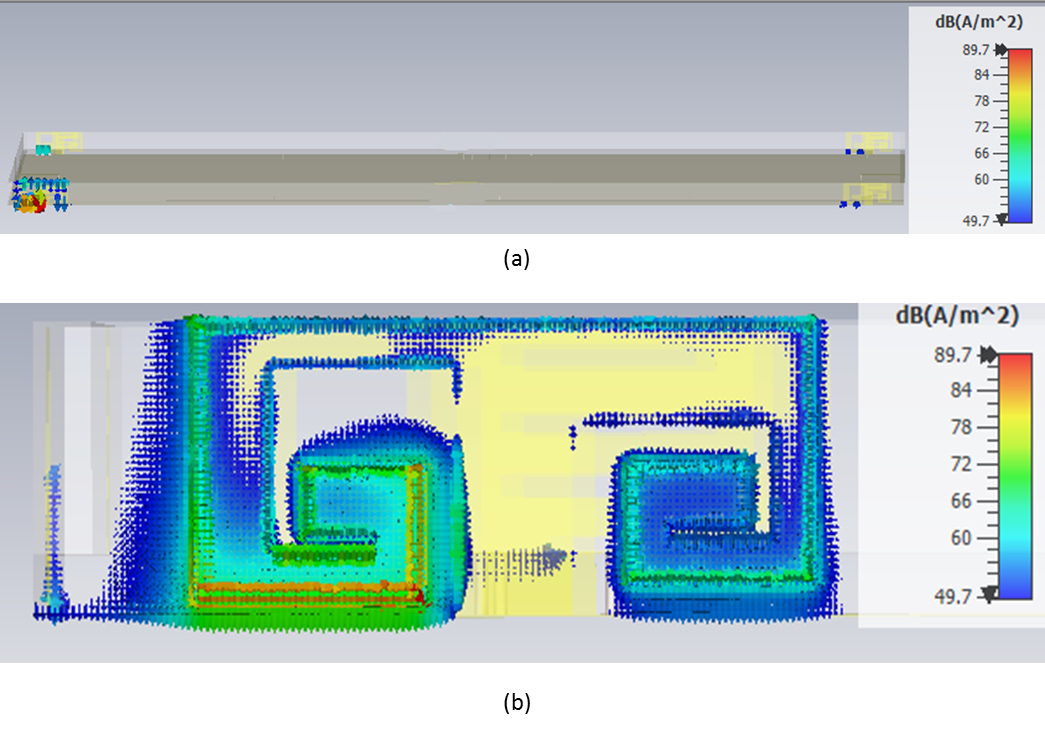

Supplement: S1 File — (ZIP) [file pone.0305060.s001.zip › supplementary information files/surface current/surface current new picture.png]

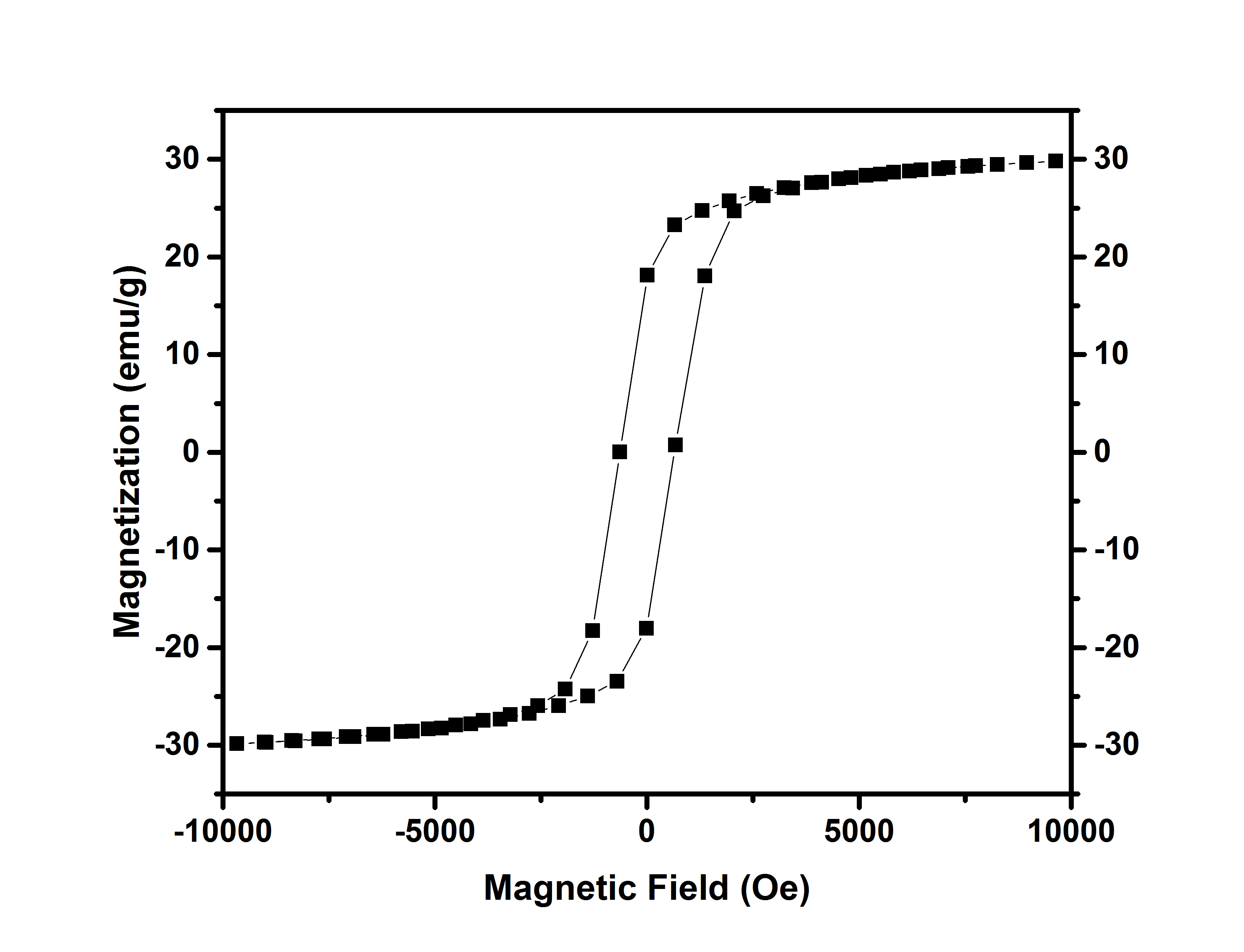

Supplement: S1 File — (ZIP) [file pone.0305060.s001.zip › supplementary information files/VSM/VSM-30-10-23.jpg]

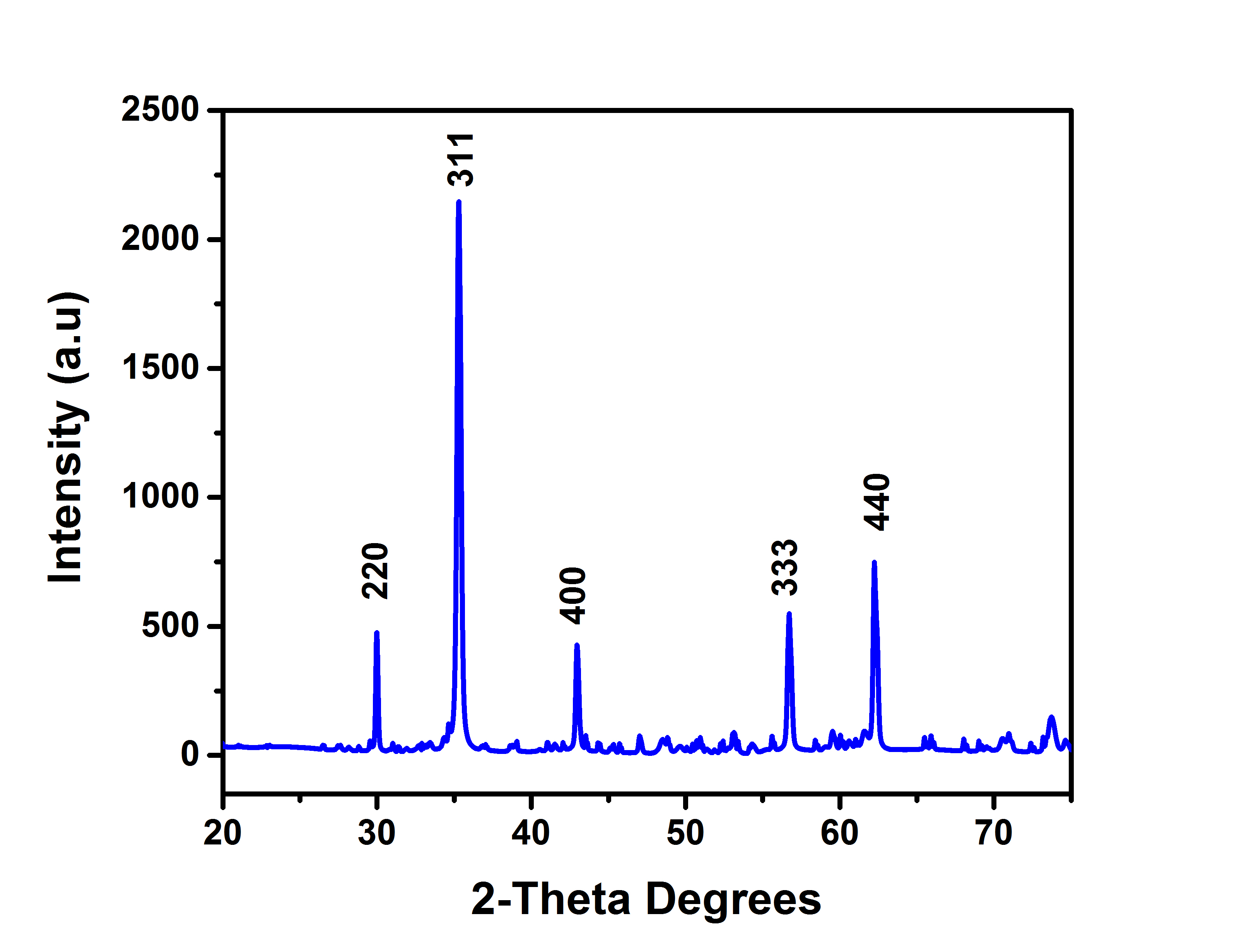

Supplement: S1 File — (ZIP) [file pone.0305060.s001.zip › supplementary information files/XRD/xrd-new for ce.jpg]
