# Supplementary material for: Synthesis and characterization of cerium doped NiZn nano ferrites as substrate material for multi band MIMO antenna
Source: PLoS One. 2024 Jul 16;19(7):e0305060. doi: 10.1371/journal.pone.0305060 (PMC11251584; doi:10.1371/journal.pone.0305060)
Supplement: Attachment — Submitted filename: Rebuttal Letter for PLOS ONE Journal.docx [file pone.0305060.s002.docx]

**Dear Editor,**

We would like to thank you first for the positive comments of our manuscript entitled “Synthesis and Characterization of Cerium doped NiZn Nano Ferrites as Substrate Material for Multi Band MIMO Antenna”. We really appreciate your help and patience. We would also like to thank the additional editor who kindly provided some technical questions, which are definitely beneficial to improving the quality of our paper. The following are our responses to yours comments. The revisions we made are highlighted in “Track Changes” in the revised submission.

We sincerely appreciate the editor’s comments on our manuscript. Some point to-point revisions were made according to the comments. The responses are stated as follows.

**Editors' comments:**

**Comment #1:**

1. Please ensure that your manuscript meets PLOS ONE's style requirements

**Response to Comment #1:**

Thanks very much for reviewer’s valuable advice. PLOS ONE’s style requirements have been followed. Following documents have been provided as per the Journal’s requirement.

1) Manuscript
2) Manuscript with tracked changes
3) Figures
4) Rebuttal letter

**Comment #2:**

2. We suggest you thoroughly copyedit your manuscript for language usage, spelling, and grammar

**Response to Comment #2:**

Thank you very much for your effort to improve the quality of our paper. Manuscript has been checked thoroughly for language, spelling and grammar and Track Changes have been highlighted in the manuscript as red.

**Comment #3:**

3. Please note that PLOS ONE has specific guidelines on code sharing for submissions in which author-generated code underpins the findings in the manuscript. In these cases, all author-generated code must be made available without restrictions upon publication of the work.

**Response to Comment #3:**

Thank you for your comment. We appreciate your concern regarding the code sharing guidelines of PLOS ONE. However, we would like to clarify that the design presented in our manuscript was prepared using CST (Computer Simulation Technology) software. While there is no specific code associated with the design, we have provided detailed descriptions of all design parameters and settings within the manuscript. These details are sufficient for the reproduction of the submitted work.

**Comment #4:**

4. We note that you have indicated that there are restrictions to data sharing for this study. PLOS only allows data to be available upon request if there are legal or ethical restrictions on sharing data publicly.

**Response to Comment #4:**

Thank you very much for your effort to improve the quality of our paper. Yes that data is available when requested.

**Comment #5:**

PLOS requires an ORCID ID for the corresponding author in Editorial Manager

**Response to Comment #5:**

Thank you very much for your effort to improve the quality of our paper. ORCID ID for the corresponding author has been created and is shares as below.

Your ORCID iD: 0009-0007-3040-5948

**Comment #6:**

Please upload a copy of Figures 1-18, to which you refer in your text on pages 9-13.

**Response to Comment #6:**

Thank you very much for your effort to improve the quality of our paper. Figures 1-18 have been uploaded separately.

**Comment #7:**

Please include captions for your Supporting Information files at the end of your manuscript, and update any in-text citations to match accordingly.

**Response to Comment #7:**

Thank you very much for your effort to improve the quality of our paper. There is no supporting information file uploaded except for the following files.

1) Manuscript
2) Manuscript with tracked changes
3) Figures
4) Rebuttal letter

**Comment #8:**

Please review your reference list to ensure that it is complete and correct

**Response to Comment #8:**

Reference list have been viewed and checked again and is found correct in all aspects.

**Additional Editor Comments:**

**Comment #1:**

There is no information regarding why crystallite size decreased with cerium composition. What is the effect of magnetic properties by doping cerium.

**Response to Comment #1:**

There is an effect of cerium doping on the crystallite size and magnetic properties of nickel-zinc (Ni-Zn) ferrites. This is explained below and highlighted as yellow in the manuscript. The same is incorporated in the manuscript and highlighted as red in the tracked changes.

“Cerium, when doped into nickel-zinc ferrites, act as a grain growth inhibitor. It impedes the movement of grain boundaries during the crystallization process, leading to smaller crystallite sizes. Cerium is known for its mixed valency, which means it can exist in both +3 and +4 oxidation states. This mixed valency can introduce additional magnetic moments in the system and modify the overall magnetic behavior of the material depending on the concentration of cerium and its distribution in the lattice [27].

Furthermore, rare-earth ion $C_{e^{3+}}$ (1.14 A˚) has bigger ionic radii as compared to$N_{i^{2+}}$,$Z_{n^{2+}}$and $F_{e^{3+}}$ions. The variations in the magnetic parameters are related to ionic radii and magnetic moments of the doping agents such as rare-earth $C_{e^{3+}}$ cation. However, the magnetic moments are noticed to be increased with the rare-earth doping which is the combined effect of the magnetic moments of all the metals cations. Bohr magneton, anisotropy constant, initial permeability, and Y-K angles depicted in table 2 confirm the tailoring of magnetic properties due to the incorporation of rare-earth $C_{e^{3+}}$ cation in NiZn ferrite.”
