# Supplementary material for: Synthesis and characterization of cerium doped NiZn nano ferrites as substrate material for multi band MIMO antenna
Source: PLoS One. 2024 Jul 16;19(7):e0305060. doi: 10.1371/journal.pone.0305060 (PMC11251584; doi:10.1371/journal.pone.0305060)
Supplement: Attachment — Submitted filename: Reviewer Remarks Cerium doped NiZn ferrite.docx [file pone.0305060.s003.docx]

The manuscript entitled, “**Synthesis and Characterization of Cerium doped 𝑵𝒊𝒁𝒏 Nano Ferrites as Substrate Material for Multi Band MIMO Antenna**” showcases structural, microstructural, dielectric, and magnetic properties for antenna application. There exist a few concerns which are stated below:

1. Revise the ‘Abstract’ section of the manuscript and avoid the sentences governing the characterization used in the manuscript in the ‘Abstract’ section.
2. The authors have used different notations for Cerium-doped Nickel Zinc Nano Ferrite throughout the manuscript.
3. Define the term ‘MIMO’ in the manuscript.
4. Include the names of the companies for the precursors used in the manuscript.
5. Include the sample preparation for dielectric studies such as pellet making etc.
6. The authors stated that “**The ferrite sample's nanocrystalline nature is confirmed. The crystal size is calculated using 108 the equation λ = 2d sin θ, where d is the size of the crystallite**” in the XRD section of the paper. Here, d is interplanar spacing, not the crystallite size and ƛ is the wavelength of the Cu K_α_ line. Correct this sentence.
7. Either use equation (1) or (2) by properly defining the terms used in expressing the relations.
8. Designation required for ‘𝐺_avg_’.
9. In the manuscript, the authors have used the term ‘samples’ but they have investigated only a single composition in the paper. If they have prepared a series of Ce-doped compositions then include the data in the manuscript.
10. Table 1 illustrates microstrain (%) for the given system. Which formula is used to calculate microstrain, if it is Williamson Hall plot analysis then include the plot for the same and mark the unit for bulk density for the sample.

Overall the manuscript requires major revision.
