# Supplementary material for: Synthesis and characterization of cerium doped NiZn nano ferrites as substrate material for multi band MIMO antenna
Source: PLoS One. 2024 Jul 16;19(7):e0305060. doi: 10.1371/journal.pone.0305060 (PMC11251584; doi:10.1371/journal.pone.0305060)
Supplement: Attachment — Submitted filename: Response to Reviewers.docx [file pone.0305060.s005.docx]

**Dear Editor,**

We would like to thank you first for the positive comments of our manuscript entitled “Synthesis and Characterization of Cerium doped $N_{i}Z_{n}$ Nano Ferrites as Substrate Material for Multi Band MIMO Antenna”. We really appreciate your help and patience. The following are our responses to yours comments. The revisions we made are highlighted in “Track Changes” in the revised submission.

Some point to-point revisions were made according to the comments and stated as follows.

**Reviewer 1 Comments:**

**Comment #1:**

1. Revise the “Abstract” section of the manuscript and avoid the sentences governing the characterization used in the manuscript in the Abstract section.

**Response to Comment #1:**

Thanks very much for reviewer’s valuable advice. Abstract section is revised and highlighted in the manuscript as tracked changes.

In addressing issues related to electromagnetic interference, the demand for ferrite materials with exceptional magnetic and dielectric properties has escalated recently. In this research, sol-gel auto combustion technique prepared Nickel zinc ferrites substituted with cerium, denoted as${Ni}_{0.5}{{Zn}_{0.5}{Ce}_{0.02}Fe}_{1.98}O_{4}$. X-ray diffraction (XRD), Vibrating Sample Magnetometer (VSM), and Field Emissions Scanning Electron Microscope (FESEM) were used to investigate the structure, magnetic properties, and morphology of Cerium doped $N_{i}Z_{n}$Nano ferrites, respectively. The magnetic and dielectric properties of the sample were examined within a frequency range of 2.5-5.5 GHz.  Sample exhibits low permittivity (2.2), high permeability (1.4), low dielectric (0.35) and magnetic loss tangent (-0.5) and highest saturation magnetization measuring 30.28 emu/g. A Novel Double-band, 4x4 MIMO window grill-modeled antenna operating on 3.5 GHz and 4.8 GHz frequency bands for 5G smartphones is designed using the CST microwave studio suite.  The performance of window grilled 4x4 MIMO antenna model with Cerium doped $N_{i}Z_{n}$ Nano ferrites as substrate, is investigated and found the return loss of -35 and -32 dB , with the bandwidth of 200MHz, gain (1.89 & 4.38 dBi), envelope correlation coefficient (0.00185), channel capacity loss (0.2bps/Hz), and inter terminal isolation of (22 & 19 dB). The results show that the antenna size is reduced with improved bandwidth, higher isolation and better diversity gain performance using Nano ferrite substrate compared to conventional dielectric substrates.

**Comment #2:**

2. The authors have used different notations for Cerium-doped Nickel Zinc Nano Ferrite throughout the manuscript.

**Response to Comment #2:**

Thank you very much for your effort to improve the quality of our paper. Same notation for Cerium doped $N_{i}Z_{n}$ Nano ferrite has been used throughout the manuscript.

**Comment #3:**

3. Define the term ‘MIMO’ in the manuscript.

**Response to Comment #3:**

Thank you very much for your effort to improve the quality of our paper. MIMO technology has been defined in the introduction section of the manuscript and highlighted in the track changes as

“MIMO (Multiple-Input Multiple-Output) is an antenna technology for wireless communications in which multiple antennas are used at both the source (transmitter) and at the destination (receiver). MIMO technology minimizes errors, optimize data speed and improve the capacity of radio transmissions by enabling data to travel over many signal paths at the same time.”

**Comment #4:**

4. Include the names of the companies for the precursors used in the manuscript.

**Response to Comment #4:**

Thank you very much for your effort to improve the quality of our paper. Names of the company is included in the manuscript and highlighted as the tracked changes.

All these initial precursor salts for the composition under study were purchased from Sigma Aldrich Company in the powder form and were of high analytical purity.

**Comment #5:**

Include the sample preparation for dielectric studies such as pellet making etc.

**Response to Comment #5:**

Thank you very much for your effort to improve the quality of our paper. Sample preparation method such as pellet making is included in the manuscript and highlighted as tracked changes.

The sintered powder ferrite sample was pressed into pallets under 25MPa pressure using a uniaxial hydraulic press. Disc shaped pellets with diameter of 14 mm and thickness of about 3 mm were obtained for dielectric measurements. In order to remove any residual moisture and densify, pallets were again sintered in an oven at a temperature of 500°C for 4 hours. These pellets were used for measuring dielectric parameters on VNA.

**Comment #6:**

The author stated that “The ferrite sample’s nanocrystalline nature is confirmed. The crystal size is calculated using 108 the equation λ = 2d sin θ, where d is the size of the crystallite” in the XRD section of the paper. Here, d is inter planar spacing, not the crystallite size and λ is the wavelength of the $C_{u}K_{\alpha}$ line. Correct this sentence.

**Response to Comment #6:**

Thank you very much for your effort to improve the quality of our paper.

The sentence was mistakenly written and corrected and highlighted in the manuscript as tracked changes.

**Comment #7:**

Either use equation (1) or (2) by properly defining the terms used in expressing the relations

**Response to Comment #7:**

Thank you very much for your effort to improve the quality of our paper. Proposed changes have been made in the manuscript and highlighted as tracked changes.

$$D={K \lambda}/{w\cos\theta}$$

(1)

Where D is the average crystallite size (in nanometers), K is the shape factor (typically assumed to be 0.9) θ is Bragg's reflection angle, 'w' represents the full-width at half maximum (FWHM) of the diffraction peak (in radians) while 'λ' denotes the wavelength of the X-rays, which is 0.154 nm.

$$a=d_{hkl}{(h^{2}+k^{2}+l^{2})}^{\frac{1}{2}}$$

(2)

The lattice parameter "a" is determined to be 8.34 Å by employing the above-mentioned equation. Table 1 shows the values of FWHM, d-spacing, lattice parameter, bulk density, X-ray density, % porosity, height (cents), micro strain, cell volume, and crystalline size.

**Comment #8:**

Designation required for “Gavg”

**Response to Comment #8:**

Thank you very much for your effort to improve the quality of our paper.

$G_{avg.}$ is the average grain size.

**Comment #9:**

In the manuscript, the authors have sued the term “samples” but they have investigated only a single composition in the paper. If they have prepared a series of Ce-doped compositions then include the data in the manuscript.

**Response to Comment #9:**

Thank you very much for your effort to improve the quality of our paper.

Only 1 composition with the formula ${Ni}_{0.5}{{Zn}_{0.5}{Ce}_{0.02}Fe}_{1.98}O_{4}$ is synthesized and reported in the manuscript. The statement “samples” was typo error and corrected in the manuscript and highlighted as tracked changes.

**Comment #10:**

Table 1 illustrates micro strain (%) for the given system. Which formula is used to calculate micro strain, if it is Williamson Hall plot analysis then include the plot for the same and mark the unit for bulk density for the sample?

**Response to Comment #10:**

Thank you very much for your effort to improve the quality of our paper. Micro strain is calculated from XRD data using Origin software and by using the relation$\varepsilon=\frac{\beta}{4\tan\theta}$. Where $\varepsilon$ is the microstrain in radians, which is the ratio of XRD peak width to the peak position. $\beta$ is the line broadening at full width half maximum (FHWM) in radians and $\theta$ is the Bragg’s angle in degrees.

**Reviewer 2 Comments:**

**Comment #1:**

The title of the paper includes the word ‘MIMO’ (Multiple Input Multiple Output) which should define within the manuscript to aid readers’ understanding.

**Response to Comment #1:**

Thank you very much for your effort to improve the quality of our paper. MIMO technology has been defined in the introduction section of the manuscript and highlighted in the track changes as

“MIMO (Multiple-Input Multiple-Output) is an antenna technology for wireless communications in which multiple antennas are used at both the source (transmitter) and at the destination (receiver). MIMO technology minimizes errors, optimize data speed and improve the capacity of radio transmissions by enabling data to travel over many signal paths at the same time.”

**Comment #2:**

The abstract section should be revised to focus solely on the objectives, methodology, and key findings of the study.

**Response to Comment #2:**

Thank you very much for your effort to improve the quality of our paper.

In addressing issues related to electromagnetic interference, the demand for ferrite materials with exceptional magnetic and dielectric properties has escalated recently. In this research, sol-gel auto combustion technique prepared Nickel zinc ferrites substituted with cerium, denoted as${Ni}_{0.5}{{Zn}_{0.5}{Ce}_{0.02}Fe}_{1.98}O_{4}$. X-ray diffraction (XRD), Vibrating Sample Magnetometer (VSM), and Field Emissions Scanning Electron Microscope (FESEM) were used to investigate the structure, magnetic properties, and morphology of Cerium doped $N_{i}Z_{n}$Nano ferrites, respectively. The magnetic and dielectric properties of the sample was examined within a frequency range of 2.5-5.5 GHz.  Sample exhibits low permittivity (2.2), high permeability (1.4), low dielectric (0.35) and magnetic loss tangent (-0.5) and highest saturation magnetization measuring 30.28 emu/g. A Novel Double-band, 4x4 MIMO window grill-modeled antenna operating on 3.5 GHz and 4.8 GHz frequency bands for 5G smartphones is designed using the CST microwave studio suite.  The performance of window grilled 4x4 MIMO antenna model with Cerium doped $N_{i}Z_{n}$ nano ferrites as substrate, is investigated and found the return loss of -35 and -32 dB , with the bandwidth of 200MHz, gain (1.89 & 4.38 dBi), envelope correlation coefficient (0.00185), channel capacity loss (0.2bps/Hz), and inter terminal isolation of (22 & 19 dB). The results show that the antenna size is reduced with improved bandwidth, higher isolation and better diversity gain performance using nano ferrite substrate compared to conventional dielectric substrates.

**Comment #3:**

The authors must address the reason for Cerium ion substitution in the ‘Introduction’ section of the manuscript by supporting references.

**Response to Comment #3:**

Thank you very much for your effort to improve the quality of our paper.

Rare-earth ions are increasingly being explored as promising additives to enhance the magnetic properties of ferrites. Among the various rare earth metals, cerium has played a crucial role in improving the characteristics of spinel ferrites.

In a recent development, [19] cobalt-zinc ferrites doped with cerium demonstrated significant absorption of the electromagnetic spectrum in the near-infrared region. [20] Suggested that cerium-doped cobalt ferrites have high potential for use in switching memory devices. Additionally, [21] investigated the structural, optical, and magnetic properties of cerium-doped $N_{i}Z_{n}$ferrites and concluded that the proposed sample is suitable for high-frequency applications.

Based on these results, the current study aims to synthesize cerium-doped $N_{i}Z_{n}$ferrite and utilize it as a substrate in the design of MIMO antennas.

**Comment #4:**

The experimental section should be revised as the permittivity and permeability were calculated for the specimen under investigation. Still the detailed procedures for sample preparation, including pellet-making techniques, should be provided for the dielectric studies.

**Response to Comment #4:**

Thank you very much for your effort to improve the quality of our paper. Experimental section has been revised and detailed procedure for sample preparation including pellet making techniques is described and highlighted in the manuscript as tracked changes.

“The sintered powder ferrite sample was pressed into pallets under 25MPa pressure using a uniaxial hydraulic press. Disc shaped pellets with diameter of 14 mm and thickness of about 3 mm were obtained for dielectric measurements. In order to remove any residual moisture and densify, pellets were again sintered in an oven at a temperature of 500°C for 4 hours. These pellets were used for measuring dielectric parameters on VNA.”

**Comment #5:**

It is advisable to include the names of the companies and purity, providing the chemicals used for the synthesis of Cerium-doped $N_{i}Z_{n}$ ferrite.

**Response to Comment #5:**

Thank you very much for your effort to improve the quality of our paper.

All these initial precursor salts for the composition under study were purchased from Sigma Aldrich Company in the powder form and were of high analytical purity.

**Comment #6:**

Explain the negative values of magnetic loss tangent in the dielectric section.

**Response to Comment #6:**

Thank you very much for your effort to improve the quality of our paper.

Negative values of tan δ indicate that the inductive behavior (μ') dominates over the resistive behavior (μ") of the sample under investigation at the given frequency range and magnetic field strength. This implies that the material exhibits more magnetic storage (inductive) properties than magnetic dissipation (resistive) properties and may be due to complex interactions between magnetic domains, hysteresis effects and domain wall movements.

**Comment #7:**

Line 153: Additionally all the samples in this study adhere to Snoek’s Law. The synthesis section didn’t show a series of Ce-doped samples.

**Response to Comment #7:**

Thank you very much for your effort to improve the quality of our paper.

Only 1 composition with the formula ${Ni}_{0.5}{{Zn}_{0.5}{Ce}_{0.02}Fe}_{1.98}O_{4}$ is synthesized and reported in the manuscript. The statement “All the samples” was typo error and corrected in the manuscript.

**Comment #8:**

Provide the designation of the terms used in the equation (1).

**Response to Comment #8:**

Thank you very much for your effort to improve the quality of our paper. Equation (1) has been properly written and highlighted as the tracked changes and mentioned below as well.

$$D={K \lambda}/{w\cos\theta}$$

(1)

, where D is the average crystallite size (in nanometers) , K is the shape factor (typically assumed to be 0.9) θ is Bragg's reflection angle, 'w' represents the full-width at half maximum (FWHM) of the diffraction peak (in radians)while 'λ' denotes the wavelength of the X-rays, which is 0.154 nm.

**Comment #9:**

Table.1 summarizes the structural parameters showing the porosity and density of the sample. However the author didn’t mention anything about these calculations in the ‘Experimental’ section of the paper. The author should explain Archimedes’ principle for the density or porosity calculations.

**Response to Comment #9:**

Thank you very much for your effort to improve the quality of our paper. Archimedes principle was used to measure the mass and volume of sample under investigation and then the density with the following relation,

$$\rho_{sample}=\frac{m_{sample}}{v_{sample}}$$

Similarly, the porosity which is the measure of the void spaces within the material obtained by using the following relation

$$\emptyset=\frac{v_{void}}{v_{sample}}$$

Where, $v_{sample}$is the total volume of the sample, $v_{void}$ is the volume of the void spaces within the sample and obtained by using Archimedes principle.

**Comment #10:**

Check relations (1) and (2) in the manuscript.

**Response to Comment #10:**

Thank you very much for your effort to improve the quality of our paper.

$$D={K \lambda}/{w\cos\theta}$$

(1)

, where D is the average crystallite size (in nanometers), K is the shape factor (typically assumed to be 0.9) θ is Bragg's reflection angle, 'w' represents the full-width at half maximum (FWHM) of the diffraction peak (in radians) while 'λ' denotes the wavelength of the X-rays, which is 0.154 nm.

$$a=d_{hkl}{(h^{2}+k^{2}+l^{2})}^{\frac{1}{2}}$$

(2)

The lattice parameter "a" is determined to be 8.34 Å by employing the above-mentioned equation. Table 1 shows the values of FWHM, d-spacing, lattice parameter, bulk density, X-ray density, % porosity, height (cents), micro strain, cell volume, and crystalline size.

**Comment #11:**

Inconsistencies exist in the notation used for Cerium-doped Nickel Zinc Nano Ferrite throughout the manuscript. The authors are recommended to use single notation.

**Response to Comment #11:**

Thank you very much for your effort to improve the quality of our paper. Same notation for Cerium doped $N_{i}Z_{n}$ Nano ferrite has been used throughout the manuscript.
