# Supplementary material for: Synthesis and characterization of cerium doped NiZn nano ferrites as substrate material for multi band MIMO antenna
Source: PLoS One. 2024 Jul 16;19(7):e0305060. doi: 10.1371/journal.pone.0305060 (PMC11251584; doi:10.1371/journal.pone.0305060)
Supplement: Attachment — Submitted filename: Reviewer comments PLOS ONE.docx [file pone.0305060.s004.docx]

The manuscript " Synthesis and Characterization of Cerium doped 𝑵𝒊𝒁𝒏 Nano Ferrites as Substrate Material for Multi Band MIMO Antenna" has been evaluated, and several concerns have been identified, which are as follows:

1. The title of the paper includes the word 'MIMO' (Multiple Input Multiple Output) which should be defined within the manuscript to aid readers' understanding.
2. The abstract section should be revised to focus solely on the objectives, methodology, and key findings of the study.
3. The authors must address the reason for Cerium ion substitution in the ‘Introduction’ section of the manuscript by supporting references.
4. The experimental section should be revised as the permittivity and permeability were calculated for the specimen under investigation. Still, the detailed procedures for sample preparation, including pellet-making techniques, should be provided for the dielectric studies.
5. It is advisable to include the names of the companies and purity, providing the chemicals used for the synthesis of Cerium-doped NiZn ferrite.
6. Explain the negative values of magnetic loss tangent in the dielectric section.
7. Line 153: ‘Additionally, all the samples in this study adhere to Snoek's Law’. The synthesis section didn’t show a series of Ce-doped samples.
8. Provide designation to the terms used in equation (1).
9. Table.1 summarizes the structural parameters showing the porosity and density of the sample. However, the authors didn’t mention anything about these calculations in the ‘Experimental’ section of the paper. The author should explain Archimedes' principle for density or porosity calculations.
10. Check relations (1) and (2) in the manuscript.
11. Inconsistencies exist in the notation used for Cerium-doped Nickel Zinc Nano Ferrite throughout the manuscript. The authors are recommended to use single notation.

Overall, significant revisions are required to address these concerns and improve the manuscript's quality, clarity, and contribution to the field of study.
